# Meta-analysis investigating the role of interleukin-6 mediated inflammation in type 2 diabetes

**DOI:** 10.1016/j.ebiom.2020.103062

**Published:** 2020-10-21

**Authors:** Nicholas Bowker, Rupal L. Shah, Stephen J. Sharp, Jian'an Luan, Isobel D. Stewart, Eleanor Wheeler, Manuel A.R. Ferreira, Aris Baras, Nicholas J. Wareham, Claudia Langenberg, Luca A. Lotta

**Affiliations:** a MRC Epidemiology Unit, University of Cambridge, Wellcome Trust-MRC Institute of Metabolic Science, Addenbrookes Hospital, Cambridge CB2 0QQ, United Kingdom; b Regeneron Genetics Center, 777 Old Saw Mill River Rd, Tarrytown, NY 10591, United States

**Keywords:** Interleukin-6, Type 2 diabetes, Genetic, Trans-ethnic

## Abstract

**Background:**

Evidence from animal models and observational epidemiology points to a role for chronic inflammation, in which interleukin 6 (IL-6) is a key player, in the pathophysiology of type 2 diabetes (T2D). However, it is unknown whether IL-6 mediated inflammation is implicated in the pathophysiology of T2D.

**Methods:**

We performed a meta-analysis of 15 prospective studies to investigate associations between IL-6 levels and incident T2D including 5,421 cases and 31,562 non-cases. We also estimated the association of a loss-of-function missense variant (Asp358Ala) in the IL-6 receptor gene (*IL6R*), previously shown to mimic the effects of IL-6R inhibition, in a large trans-ethnic meta-analysis of six T2D case-control studies including 260,614 cases and 1,350,640 controls.

**Findings:**

In a meta-analysis of 15 prospective studies, higher levels of IL-6 (per log pg/mL) were significantly associated with a higher risk of incident T2D (1·24 95% CI, 1·17, 1·32; *P* = 1 × 10^−12^). In a trans-ethnic meta-analysis of 260,614 cases and 1,350,640 controls, the *IL6R* Asp358Ala missense variant was associated with lower odds of T2D (OR, 0·98; 95% CI, 0·97, 0·99; *P* = 2 × 10^−7^). This association was not due to diagnostic misclassification and was consistent across ethnic groups. IL-6 levels mediated up to 5% of the association between higher body mass index and T2D.

**Interpretation:**

Large-scale human prospective and genetic data provide evidence that IL-6 mediated inflammation is implicated in the etiology of T2D but suggest that the impact of this pathway on disease risk in the general population is likely to be small.

**Funding:**

The EPIC—Norfolk study has received funding from the Medical Research Council (MRC) (MR/N003284/1, MC-UU_12015/1 and MC_PC_13048) and Cancer Research UK (C864/A14136). The Fenland Study is funded by the MRC (MC_UU_12015/1 and MC_PC_13046).

**Research in context**

**Evidence before this study**

Chronic inflammation, in which interleukin 6 (IL-6) is a key player, has been hypothesised to play a role in the pathophysiology of type 2 diabetes (T2D), largely on the basis of experimental evidence from animal models. In line with this hypothesis, IL-6 levels are associated with obesity, insulin resistance and type 2 diabetes risk in observational epidemiology studies. However, it is unclear whether there is a genetic association between variants in the IL-6 receptor gene (*IL6R*) and type 2 diabetes risk. If so, then blocking IL-6 mediated inflammation via the IL6R may help prevent or treat type 2 diabetes.

**Added value of this study**

We conducted a large trans-ethnic meta-analysis of the association with type 2 diabetes of the *IL6R* genetic variant Asp358Ala, an experimentally validated loss of function variant in the IL-6 receptor previously shown to mimic the effects of IL-6R inhibition therapy. In an analysis of 260,614 cases and 1350,640 controls, we find a statistically robust association between the Asp358Ala and lower risk of type 2 diabetes (OR, 0·98; 95% CI, 0·97, 0·99; *P* = 2 × 10^−7^), which is consistent across ancestries and not due to diagnostic misclassification with type 1 diabetes. Using both observational and genetic mediation analyses, we show that the impact of the IL-6 pathway on risk of type 2 diabetes due to obesity is modest (3–5%), which challenges a prevailing hypothesis that IL-6 has an important role in the link between obesity and diabetes risk.

**Implications of all the available evidence**

IL-6 mediated inflammation is likely to be implicated in type 2 diabetes, but evidence from this study suggests that the contribution of this pathway to the risk of type 2 diabetes in the general population is likely to be small.

Alt-text: Unlabelled box

## 1 Introduction

Chronic inflammation, which is partly mediated via the interleukin 6 (IL-6) pathway, has been hypothesized to play a critical role in the pathophysiology of type 2 diabetes (T2D) [1,2]. In obesity, the enhanced production of IL-6 and other pro-inflammatory mediators as a result of higher BMI is associated with insulin resistance [3], [4], [5] and with a higher risk of T2D [6,7] and coronary artery disease [8]. In addition, evidence from animal models and observational epidemiology has linked chronic inflammation with metabolic disease risk factors including disruption of lipid metabolism, [5,9] hypertension [10], [11], [12] and impaired insulin secretion [1,3,13,14]. On this basis, it has been hypothesised that therapeutically targeting inflammatory pathways may reduce the risk of T2D or improve glycaemic control in people with diabetes [2,7,15,16].

While small scale clinical trials had provided initial supportive evidence of possible benefit, [1,17] this notion has been strongly challenged by the negative findings of the Canakinumab Anti-inflammatory Thrombosis Outcome Study [18,19] (CANTOS). In a pre-specified secondary analysis of this trial of 10,061 people with previous myocardial infarction and high C-reactive protein (CRP), treatment with the interleukin-1β inhibitor canakinumab substantially reduced circulating inflammatory markers, but did not significantly reduce risk of new-onset diabetes in participants without diabetes at recruitment, or improve glycaemia in participants with diabetes [20].

However, the possible therapeutic benefit on diabetes risk of directly inhibiting the IL-6 pathway has not been directly evaluated in large trials. In cardiovascular medicine, biomarker studies showing associations of higher IL-6 levels with higher cardiovascular disease incidence [8,[21], [22], [23]] and genetic studies [24], [25], [26] showing robust associations of an experimentally-validated [27] partial loss-of-function variant in the IL-6 receptor gene with protection against coronary disease have provided the evidence base for ongoing clinical trials that are evaluating the effects of IL-6 receptor inhibition in myocardial infarction.

In this study, biomarker and human genetic data from large-scale population-based cohorts were combined to investigate the possible etiologic role of IL-6 and IL-6 receptor (IL-6R) related pathways in T2D risk.

## 2 Methods

### 2.1 Study design

**Fig. 1:**
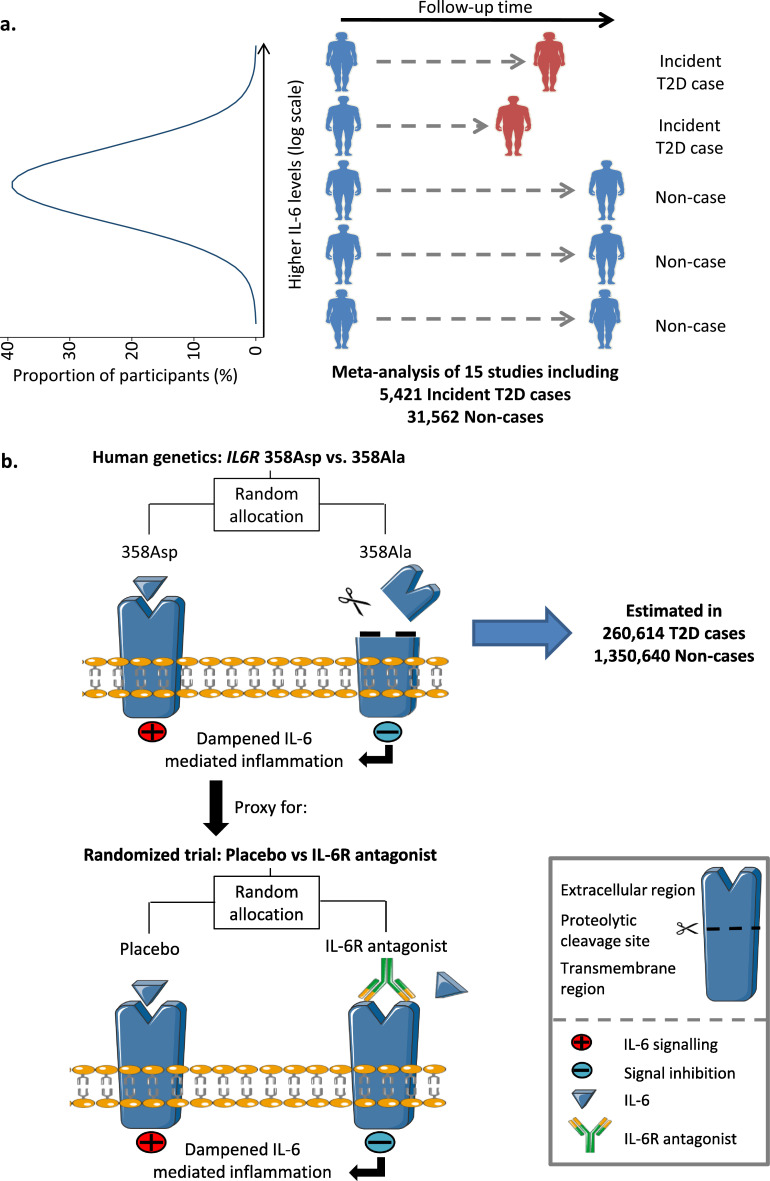
Study design. Panel a. Prospective association between IL-6 levels and risk of incident type 2 diabetes. Panel b. Mechanism by which *IL6R* Asp358Ala leads to dampened IL-6 mediated inflammation. Asp358Ala increases the proteolytic cleavage of the IL-6R, depicted with a pair of scissors, resulting in dampened classical IL-6 signaling. This is similar to the effects of an IL-6R antagonist, shown at the bottom of the panel. Abbreviations: T2D, type 2 diabetes; IL-6, interleukin-6; IL-6R, interleukin 6 receptor (protein); Asp, aspartic acid; Ala, alanine; gp130, Glycoprotein 130.Modified from Servier Medical Art, licensed under a Creative Commons Attribution 3·0 Generic License from http://smart·servier·com.

Three sets of analyses were used to investigate the relationships between the IL-6 pathway and T2D (Table 1 and Fig. 1). In stage 1, the associations between IL-6 levels, glycaemic and anthropometric traits, and risk of incident T2D were estimated in two population-based cohort studies and a meta-analysis of 15 prospective studies (Fig. 1a, Table 1 and eTable 1). In stage 2, the association of a missense variant (Asp358Ala) in the interleukin 6 receptor gene (*IL6R*) with T2D was estimated in a trans-ethnic meta-analysis of six case-control studies (Fig. 1b, Table 1 and eTable 1). In stage 3, the proportion of the association between body mass index (BMI) and risk of cardio-metabolic disease outcomes mediated by IL-6 levels was estimated in three population-based cohort studies (eFig. 1**,** Table 1 and eTable 1). All studies were approved by local institutional review boards and ethics committees and participants gave written informed consent.

**Table 1:** Summary of the study design·.

Stage and Aim	Independent Variables	Primary Outcome Variables	Outcome Data Sources	Outcome measure and statistical significance
Stage 1: Estimate the association of IL-6 levels with incident type 2 diabetes	IL-6 levels	Type 2 diabetes	EPIC—Norfolk (individual-level data); 14 prospective studies (summary statistics)	Risk ratio and 95% CI *P* < ·05
Stage 2: Estimate the association of *IL6R* Asp358Ala with type 2 diabetes	Genotypes of Asp358Ala	Type 2 diabetes	UK Biobank (individual-level data); DIAMANTE [34], MVP [26], Finngen [36], Geisinger [37], Suzuki et al.*·*[39] (summary statistics)	Odds ratio and 95% CI *P* < ·05
Stage 3: Estimate the proportion of the associations between BMI and cardiometabolic disease and BMI and fasting insulin that are mediated by IL-6	Independent variable: BMI (observational); polygenic score for BMI (genetic) Mediator: IL-6 levels	Type 2 diabetes, coronary artery disease and fasting insulin levels	EPIC—Norfolk, UK Biobank, Fenland (individual-level data) and MAGIC (summary data)	Mediation percentage and 95% CI (%)

Abbreviations: IL-6, interleukin 6; EPIC, European prospective investigation into cancer and nutrition; DIAMANTE; Diabetes meta-analysis of trans-ethnic association studies; MVP, Million veteran program; CI, confidence interval.

Data sources for secondary analyses are summarised in eTable 1.

### 2.2 Studies and participants

EPIC—Norfolk [28] (data collection: 1998–2019; Table 1) is a population-based prospective cohort of individuals aged between 40 and 79 years and living in Norfolk (a county of the United Kingdom) at the time of recruitment from primary-care outpatient clinics in the city of Norwich and surrounding areas. Participant selection was based on two criteria, those who attended a visit between 1998 and 2000 and had available blood samples for IL-6 measurements were eligible for inclusion in this study. The study was approved by the Norfolk Research Ethics Committee (ref. 05/Q0101/191) and all participants gave their written consent before entering the study.

Fenland [29] (data collection: 2005–2019; Table 1) is a population-based cohort study of individuals without diabetes who were born between the years of 1950 and 1975 and recruited through population-based general practice registers in Cambridge, Ely and Wisbech (Cambridgeshire county, United Kingdom). Participant selection was based on two criteria, those who attended a visit between 2005 and 2015 and had available blood samples for IL-6 measurements were eligible for inclusion in this study. Ethical approval for the study was given by the Cambridge Local Ethics committee (ref. 04/Q0108/19) and all participants gave their written consent prior to entering the study.

UK Biobank [30] (data collection: 2006–2019; Table 1) is a population-based cohort study of individuals recruited from 22 rural and urban recruitment centres in the United Kingdom. European ancestry participants with available genome-wide genotyping and phenotypic data were included in this study. Ethical approval for the UK Biobank study was given by the North West - Haydock Research Ethics Committee (16/NW/0274). This research was conducted using application 44448. Participants gave their electronic consent to use their anonymised data and samples for health-related research, to be re-contacted for further sub-studies, and for access to their health-related records.

The association of IL-6 levels with incident T2D was estimated in a meta-analysis of prospective studies including EPIC—Norfolk [28] and 14 other studies [7,[31], [32], [33]] (data collection: 1998–2018) as described in eMethods 1 and eTables 1–4. Estimates from EPIC—Norfolk were generated as part of this study and contributed 8% of the total incident cases and 22% of the non-cases to the analysis. The association of *IL6R* Asp358Ala with prevalent T2D was estimated in a trans-ethnic meta-analysis of results from individual-level data in UK Biobank, and summary-level data from the European ancestry analysis of the Diabetes Meta-analysis of Trans-ethnic Association Studies [34] (DIAMANTE; data collection: 2018–2019; eTable 1), Million Veteran Program [26,35] (MVP; data collection: 2011–2019; eTable 1), Finngen [36] (Finngen; data collection: 1996–2016; eTable 1), MyCode Study from the DiscovEHR Collaboration between Regeneron Genetics Center and Geisinger Health System [37,38] (Geisinger; data collection: 2007–2020; eTable 1) and a recent Japanese meta-analysis [39] (Suzuki et al.·; data collection: 2003–2012; eTable 1). The association of *IL6R* Asp358Ala with prevalent type 1 diabetes was estimated in a meta-analysis of results from individual-level data in UK Biobank, and summary-level data from the Type 1 Diabetes Genetics consortium [40] (T1DGC; data collection: 2004–2019; eTable 1) and MVP [35] (data collection: 2011–2019; eTable 1). Summary-level results from 10 previously published genome-wide association studies were used in different analyses of the study (eTable 1).These included associations with BMI, BMI-adjusted waist-to-hip ratio (WHR), unadjusted WHR, waist- and hip-circumference from a meta-analysis of Genetic Investigation of Anthropometric Traits [41] (GIANT) consortium and UK Biobank [42], associations with fasting glucose, glucose at 2 h after an oral glucose challenge and fasting insulin from the Meta-analyses of Glucose and Insulin-related Traits consortium [43], [44], [45] (MAGIC).The association with HbA1c was estimated in a meta-analysis of UK Biobank [30] and MAGIC [46] (eTable 1).The association with non-fasting glucose was estimated in UK Biobank [30].

### 2.3 Exposure and outcome variables

In stage 1, the exposure variable was the circulating level of IL-6 (log-transformed and expressed in log-pg/mL) measured in plasma samples using a high-performance electrochemiluminescence immunoassay (Meso Scale Discovery®, Rockville, MD, USA).

The outcome variable used in the meta-analysis was incident T2D from the EPIC—Norfolk study. Fasting glucose, glucose at 2 h after an oral glucose challenge, HbA1c, fasting insulin, waist-to-hip ratio (WHR), BMI, iron, transferrin, ferritin, body fat percentage, abdominal fat mass, gluteofemoral fat mass, leg fat mass, abdominal to gluteofemoral fat mass ratio, peripheral fat mass, visceral fat mass and subcutaneous fat mass from the Fenland study were used in observational analyses. Incident T2D was defined as new-onset T2D in a participant without diabetes when IL-6 levels were measured, adjudicated on the basis of (a) a hospital admission record or mortality registry record of “type 2 diabetes mellitus” (International Statistical Classification of Diseases and Related Health Problems Tenth Revision [ICD-10] code E11); or (b) self-reported physician diagnosis of T2D during a follow-up visit; or (c) a value of HbA1c above 6·5% (48 mmol/mol) at a follow-up visit. Participants with T2D at the time of IL-6 measurement were excluded. Fasting glucose, fasting insulin and HbA1c were the values of circulating glucose (in mmol/L), insulin (natural log-transformed and expressed in log-pmol/L) and glycated hemoglobin (in%) measured in whole blood after overnight fasting. Two-hour glucose was the value of glucose (in mmol/L) measured in plasma two-hours after a 75-gram oral glucose challenge. Glucose, iron (in µmol/L), transferrin (in g/L) and ferritin (in µg/L) were measured using the Dimension RxL Integrated Chemistry System (Siemens, Germany). Weight was measured to the nearest 200 grams using a calibrated electronic scale (TANITA model BC-418 MA; Tanita, Tokyo, Japan). Height was measured to the nearest 0·1 cm with a wall-mounted stadiometer (SECA 240; Seca, Birmingham, United Kingdom). BMI (in kg/m^2^) was calculated as weight divided by height squared. Waist and hip circumferences were measured to the nearest 0·1 cm with a non-stretchable fiber-glass insertion tape (D loop tape; Chasmors Ltd, London, United Kingdom). WHR was the ratio between the waist and hip circumferences. Compartmental fat masses were measured in grams by Dual-energy X-ray absorptiometry (DEXA), a whole-body, low-intensity X-ray scan that precisely quantifies fat mass in different body regions. DEXA scans were performed using a Lunar Prodigy advanced fan beam scanner (GE Healthcare, Bedford, UK) according to a standard procedure (eMethods 1 and eTable 5).

In stage 2, the exposure variable was the genotype of rs2228145 (HUGO Gene Nomenclature Committee gene name, *IL6R*; transcript change, NCBI transcript identifier NM_000565·3 c·1073A>*C*; protein change, Asp358Ala). The variant 358Ala protein (encoded by the minor allele C; allele frequency in European ancestry participants in the 1000 Genomes Project, 36%) has been experimentally shown to be more susceptible to proteolytic cleavage by ADAM-proteases [47], resulting in enhanced shedding of soluble IL-6R, reduced expression of the membrane-bound form of the receptor [27] and impaired cellular responsiveness to IL-6 [27]. On this basis, the variant allele has been hypothesized to impair signaling of the IL-6 receptor via its classical pathway in a way that mimics the effects of IL-6R inhibition using therapeutic monoclonal antibodies approved for the treatment of autoimmune conditions [24,25]. The pattern of association of this variant with a variety of intermediate traits and outcomes is highly consistent with the effects of IL-6R inhibitory drugs on those same traits in randomized clinical trials [24,25]. Hence, this variant has been used in several previous studies as a genetic instrument to study the likely consequences of pharmacological IL-6R inhibition. In UK Biobank, the variant genotype was obtained via imputation to the Haplotype Reference Consortium v1·1, [48] UK10K [49] and 1000 genomes phase 3 [50] reference panels as previously described. The imputation quality score was 1 (i.e. the maximum possible score), indicating excellent imputation quality comparable with direct genotyping.

The outcome variables were T2D in the primary analysis and type 1 diabetes, fasting glucose, 2-hour glucose, non-fasted glucose, fasting insulin, HbA1c, BMI, WHR, BMI-adjusted WHR, waist and hip circumference in secondary analyses. In UK Biobank, binary definitions of prevalent disease and a case-control analytical design were used as previously described [42]. Participants were classified as cases of T2D if they had: (1) self-reported T2D diagnosis or (2) self-reported use of oral anti-diabetic medications at nurse interview or at digital questionnaire, or (3) an electronic health record consistent with T2D (ICD-10 code E11). Participants were classified as cases of type 1 diabetes if they had: (1) self-reported type 1 diabetes diagnosis or (2) self-reported use of insulin but not oral anti-diabetic medications at nurse interview or at digital questionnaire, or (3) an electronic health record consistent with type 1 diabetes (ICD-10 code E10). Participants with evidence of diabetes but for whom the type of diabetes was unclear (e.g. participants reporting T2D but treated with insulin only or participants with electronic health records of both type 1 and T2D, code E10 in some electronic records but E11 in other records) were excluded. Controls were participants who (1) did not self-report a diagnosis of diabetes of any type, and (2) did not take any diabetes medications, and (3) did not have an electronic health record of diabetes of any type. Summary results for the association with glycaemic and anthropometric traits were extracted from the results of previous genome-wide association studies (eMethods 2, eTable 1 and eTable 6).

In stage 3, the exposure variables were BMI and a polygenic score for higher BMI. The latter was derived using 97 BMI-associated common variants from a previous genome-wide association study [51] (eTable 7). For each individual, the polygenic score was computed by adding the number of copies of each contributing genetic variant weighted by its association estimate in kg/m^2^ units of BMI per allele.

The outcome variables were T2D, coronary artery disease and fasting insulin levels. T2D was defined as described above. In EPIC—Norfolk, incident coronary artery disease was defined as new-onset coronary artery disease in a participant without coronary artery disease at the time of IL-6 measurement based on an electronic health record consistent with ischemic heart disease (ICD-10 codes I20–25). Participants with coronary artery disease at the time of IL-6 measurement were excluded. In UK Biobank, prevalent coronary artery disease was defined as either (1) myocardial infarction or coronary disease documented in the participant's medical history at the time of enrolment by a trained nurse or (2) an electronic health record of acute myocardial infarction or its complications (ICD-10 codes I21-I23). Controls were participants who did not meet any of these criteria. Summary-level results from MAGIC [45] were used for fasting insulin levels.

The mediator variable was the level of IL-6 in plasma measured by electrochemiluminescence as described above.

### 2.4 Statistical analysis

In stage 1, Cox regression models were used to estimate the association between IL-6 levels and T2D. Age, sex, BMI, WHR, ethnicity, education level, family history of T2D, smoking status, average units of alcohol per week and average self-reported level of physical activity were covariates in the models. Incident rates were calculated as number of incident cases divided by the total person-years of follow-up in people at risk. Absolute rate differences were obtained for each decile of the IL-6 distribution in EPIC—Norfolk relative to bottom decile (i.e. lowest IL-6 level) and 95% confidence intervals were calculated as ± 1·96 * √(number of incident cases in decile/(total follow-up in decile)  + number of incident cases in bottom quantile/(total follow-up in bottom decile)). The interaction between IL-6 levels and BMI on risk of incident type 2 diabetes was estimated by including an interaction term for BMI in the Cox regression model and adjusting for covariates as before, with the exclusion of BMI as a covariate. Estimates of association from the EPIC—Norfolk study and other 14 prospective studies [7,[31], [32], [33]] were pooled using inverse-variance weighted fixed-effect meta-analysis, making the assumption that the study-specific estimates of association all approximated the relative risk. Linear regression models were used to estimate associations between IL-6 and glycaemic traits. Models were adjusted using three methods: (1) for age and sex, (2) for age, sex and DEXA-derived detailed anthropometric variables, (3) for age, sex, DEXA variables and iron variables (iron, ferritin and transferrin) to mitigate the effects of IL-6 levels on hepcidin and iron metabolism.

In stage 2, the association between *IL6R* Asp358Ala and IL-6 levels in Fenland and EPIC—Norfolk were estimated using linear regression adjusting for age, sex, genotyping platform and the first 10 genetic principal components. The associations of *IL6R* Asp358Ala in UK Biobank were estimated using linear (continuous outcomes) or logistic (binary outcomes) regression. To minimize genetic confounding, association analyses were restricted to European ancestry individuals, identified by combining k-means clustering of genetic principal components with self-reported ancestry. To control for relatedness, analyses were either clustered using family structure data (third degree relatives) and adjusted for 40 genetic principal components or performed using linear mixed-effects models adjusting for a genomic kinship matrix. All analyses were adjusted for age, sex and genotyping array. Association estimates from UK Biobank and other studies were pooled using inverse-variance weighted fixed-effect meta-analysis.

Various secondary and sensitivity analyses, described below, were conducted to provide context for the association of *IL6R* Asp358Ala with T2D. In UK Biobank, the proportion of variance in T2D and coronary artery disease explained by the *IL6R* Asp358Ala variant was estimated as the difference in R^2^ between a regression model including Asp358Ala and covariates (age, sex, genotyping array and genetic principal components) and one including only the covariates. The heritability explained by the variant was estimated by rescaling the variance explained by the heritability in T2D and coronary artery disease estimated using linkage disequilibrium score regression [52] (eMethods 2). The association of Asp358Ala with T2D was also estimated while conditioning on an independent association signal upstream of Asp358Ala.

To assess whether the association with T2D might be influenced by misclassification of cases of type 1 diabetes (i.e. an etiologic subtype of diabetes where immune pathways have an established causal role) as cases of T2D, the association of *IL6R* Asp358Ala with T2D was tested in individuals below the median value of a polygenic score for type 1 diabetes [53]. This approach has been shown to enable the exclusion of over 95% type 1 diabetes cases present in a dataset (sensitivity of values above the median to detect type 1 diabetes, 96%; false negative rate [i.e. type 1 diabetes cases left in individuals below the median], 4%) [53]. Simulations revealed that, when using this approach, only 46 (0·4%) misclassified cases out of over 11,000 cases of T2D would remain in the analysis, even in the extreme scenario that 100% of type 1 diabetes cases in UK Biobank were misclassified as T2D (eMethods 2).

To assess whether genetic predisposition to lower IL-6R signaling due to *IL6R* Asp358Ala genotypes interact with genetic predisposition to known risk factors for T2D, the association of *IL6R* Asp358Ala with T2D was estimated in individuals above or below the median value for 10 polygenic scores capturing genetic predisposition to higher fasting glucose [44,45], fasting insulin [44,45], HbA1c via glycaemic-related mechanisms [46], insulin resistance [44,54], impaired insulin secretion [54], BMI [41], BMI-adjusted WHR [42], BMI-adjusted WHR via lower gluteofemoral fat [42], BMI-adjusted WHR via higher abdominal fat [42] and lower BMI via a β-arrestin biased gain-of-function variants in *MC4R* [55] (eTable 7). The analysis was then repeated using HbA1c levels in UK Biobank as the outcome and a polygenic score for higher BMI as the interaction variable.

While both experimental [27] and genetic [24,25] association studies have shown that rs2228145-C (358Ala) has phenotypic associations that mimic the effects of IL-6R inhibitory therapy, an association of this variant with lower risk of T2D does not necessarily mean that IL-6R inhibitory therapy will produce clinically-meaningful reduction in the risk of T2D. Even when assuming that the effects of a genetic variant and that of a drug on the target are qualitatively the same, two differences remain. The first is that genetic variants are usually associated with small differences in the activity of the target gene, while drugs usually have large effects on target activity. The second is that the small differences in target gene activity associated with a genetic variant are lifelong, while the effect of drugs is usually assessed in trials of short duration. Statistical approaches have been proposed to model these differences and formulate approximate projections of a range of possible effects of drug treatment on the basis of the magnitude of genetic associations [56,57]. In this study, a projected range of possible effects for IL-6R blocking therapy on incident T2D in a primary prevention setting were formulated using (a) the genetic association of *IL6R* Asp358Ala with diabetes in this study, (b) the genetic association of *IL6R* Asp358Ala with C-reactive protein (used as biomarker of IL-6R mediated inflammation) in this and other studies [25] and (c) the effects of IL-6R blocking therapy on C-reactive protein (the most-widely used biomarker of target engagement for IL-6R blocking therapy) in randomized clinical trials [24]. Assumptions and details of the methods employed in these calculations are in eMethods 2.

In stage 3, two mediation models were utilized (eFig. 1). The first estimated the proportion of the association between measured BMI and cardio-metabolic disease outcomes mediated by IL-6 levels (observational mediation). The second estimated the proportion of the association between genetic predisposition to higher BMI and cardio-metabolic disease outcomes mediated by IL-6 levels (genetic mediation). The genetic analysis was also repeated using fasting insulin levels as the outcome. As genetic associations are robust to non-genetic forms of confounding, we hypothesized that the second model would be more conservative in estimating the role of IL-6 levels as a possible mediator.

The proportion of association mediated by IL-6 was estimated using the Baron-Kenny [58] method. To obtain a 95% confidence interval for this proportion, 80% of individuals from each sample population used in each step of the mediation analysis were randomly sampled and the respective analyses were performed with 1000 iterations per arm of the framework. Sampled individuals were replaced prior to the next iteration. Mediation estimates were calculated for the 1000 iterations and the 2·5th and 97·5th percentile mediation estimates respectively were used as the lower and upper bounds of the 95% confidence interval.

P-values <0·05, or the Bonferroni correction corresponding to *P*<·05 for each analysis of the study with multiple exposures or outcomes, were considered statistically significant. All reported P-values were from 2-tailed statistical tests. Statistical analyses were performed using STATA v14·2 (StataCorp, College Station, Texas 77,845 USA), R v3·2·2 (The R Foundation for Statistical Computing), BOLT-LMM v2·3·2 [59] and METAL v2011–03–25 [60].

## 3 Role of the funding source

The funding source for this study had no role in study design, data collection, data analysis, data interpretation, or in the writing of the manuscript. The corresponding author had full access to all data used in the study and had final responsibility for the decision to submit for publication.

## 4 Results

### 4.1 IL-6 levels, anthropometric and glycaemic measures and risk of T2D

**Fig. 2:**
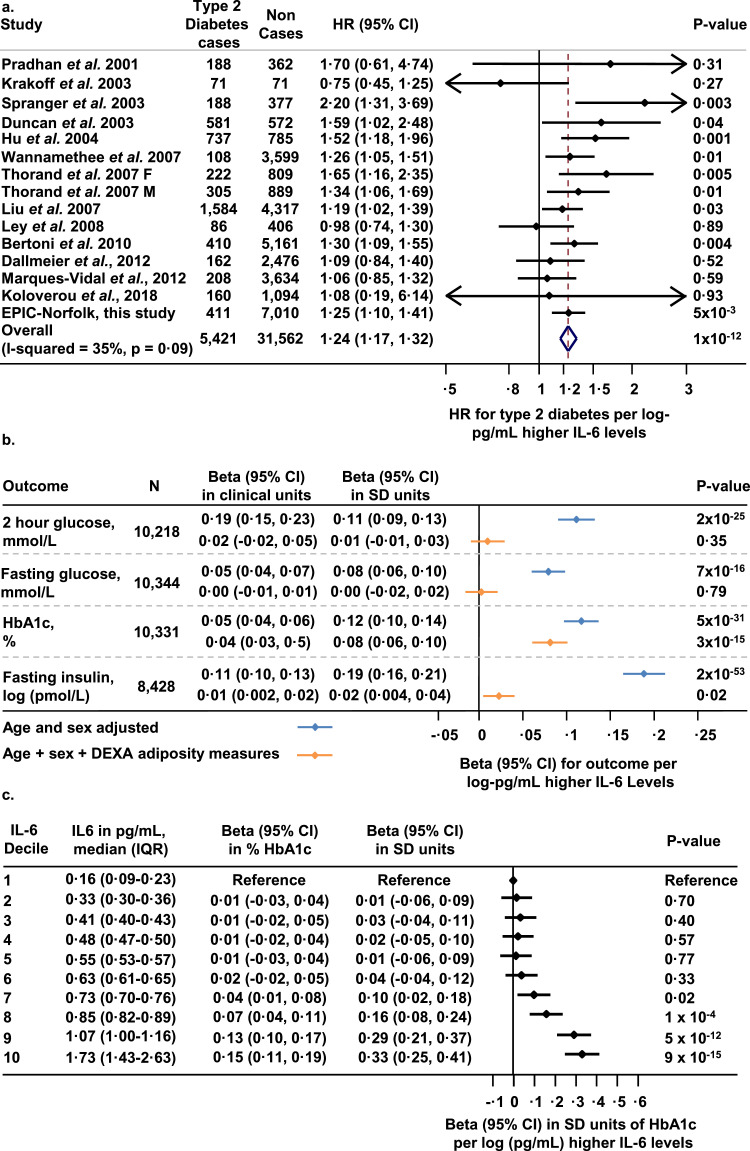
Association of IL-6 levels with type 2 diabetes and glycaemic traits. Panel a. Association between higher IL-6 levels and type 2 diabetes in 15 prospective studies. The study-specific estimates of association (here labelled “Hazard Ratio” (HR)) and their 95% confidence intervals are shown per log-pg/mL higher IL-6 levels. Thorand et al.*·*, 2007 is depicted with sex-stratified estimates as different IL-6 quantitation methods were used in men and women. Panel b. Association between IL-6 levels and glycaemic traits in the Fenland study. Estimates are presented in standard deviation and clinical units of continuous outcome per log-pg/mL higher IL-6 levels. Panel c. Association between IL-6 levels (in deciles) and HbA1c.Estimates are shown for a given decile compared to the bottom decile (reference group) and are adjusted for age, sex, DEXA adiposity measures and iron-related traits. Abbreviations: HR, hazard ratio; CI, confidence interval; F, females; M, males; pg, picograms; mL, millilitres; L, litres, mmol; millimoles; pmol, picomoles; HbA1c, glycated hemoglobin; DEXA, dual-energy X-ray absorptiometry; IQR, inter-quartile range; SD, standard deviation.

Among 7421 participants of the EPIC—Norfolk cohort, median levels of IL-6 were 0·59 pg/mL (interquartile range [IQR], 0·42, 0·85 pg/mL; Table 2). A total of 411 incident cases of T2D occurred over 122,115 person-years of follow-up (incidence rate, 3·4 per 1000 person-years). Higher levels of IL-6 were associated with higher incidence of T2D (hazard ratio [HR] per log-pg/mL higher IL-6 levels, 1·25; 95% CI, 1·10, 1·41; *P*<·001; Cox regression), with participants in the top decile of IL-6 levels having approximately 2-fold higher hazard compared to the bottom decile (HR, 2·14; 95% CI, 1·21, 3·81; *P* = 009; Cox regression; absolute rate difference in incident cases per 1000 person-years, 4·2; 95% CI, 2·8, 5·7; eFigure 3). For comparison, participants in the top decile of BMI had approximately 6-fold higher hazard compared to those in the bottom decile (HR, 5·64; 95% CI, 2·77, 11·49; *P* = 2 × 10^−6^; Cox regression; eFigure 4). No evidence of an interaction between IL-6 levels and BMI was found on incident type 2 diabetes risk (P_interaction_=0·86; Cox regression). A meta-analysis of 15 prospective studies including 5421 incident cases and 31,562 non-cases showed a robust association between higher IL-6 levels and higher incident T2D, with a pooled hazard ratio that was approximately the same as that of the EPIC—Norfolk analysis (Fig. 2a). In sensitivity analyses, no evidence of publication bias or bias induced via differing IL-6 quantitation methods used between studies was found (P_Egger_ = 0·44; Egger test; eMethods 1). In 10,344 participants of the Fenland cohort, IL-6 levels were positively correlated with DEXA-measures of overall adiposity and abdominal fat distribution, which accounted for 9% of the variance in IL-6 levels (eTable 8). After extensive adjustment for adiposity measures, higher IL-6 levels remained associated with higher HbA1c levels, but not with fasting glucose, 2-hour glucose or fasting insulin (Figs. 2B and 2C). This remained after further adjustment for iron-related traits to mitigate the effects of IL-6 on hepcidin and iron metabolism (beta in% per log-pg/mL higher IL-6 levels, 0·04; 95% CI, 0·03, 0·05; *P*<·001; linear regression).

**Table 2:** Study participants·.

Study	Fenland	EPIC—Norfolk	UK Biobank
**Participants, N**	10,344	7421	447,491
**Age at baseline, mean years (SD)**	48 (8)	62 (9)	57 (8)
**Women, N (%)**	5528 (53)	4536 (61)	243,114 (54)
**Men, N (%)**	4816 (47)	2885 (39)	204,377 (46)
**Current smokers, N (%)**	1236 (12)	574 (8)	46,459 (10)
**BMI in kg/m^2^, mean (SD)**	26·7 (4·5)	26·4 (3·8)	27·4 (4·7)
**Waist-to-hip ratio, mean (SD)**	0·88 (0·09)	0·84 (0·09)	0·87 (0·09)
**Systolic blood pressure in mmHg, mean (SD)**	122 (15)	134 (18)	138 (19)
**Diastolic blood pressure in mmHg, mean (SD)**	74 (10)	81 (11)	82 (10)
**IL-6 in pg/mL, median (IQR)**	0·51 (0·34, 0·74)	0·59 (0·42, 0·85)	N/A
**IL-6 in log-pg/mL, mean (SD)**	−0·77 (0·94)	−0·55 (0·83)	N/A

In UK Biobank genotyping was performed using the Affymetrix 500 K and Affymetrix UK BiLEVE genotyping chips and imputation was performed using the Haplotype Reference Consortium v1·1, UK10K and 1000 Genomes phase 3 reference panels.IL-6 levels were not measured in UK Biobank.

Abbreviations: N/A, not available; N, number of participants; SD, standard deviation; BMI, body mass index; IL-6, Interleukin-6; IQR, Interquartile range.

### 4.2 Genetically impaired IL6R signaling and T2D risk

**Fig. 3:**
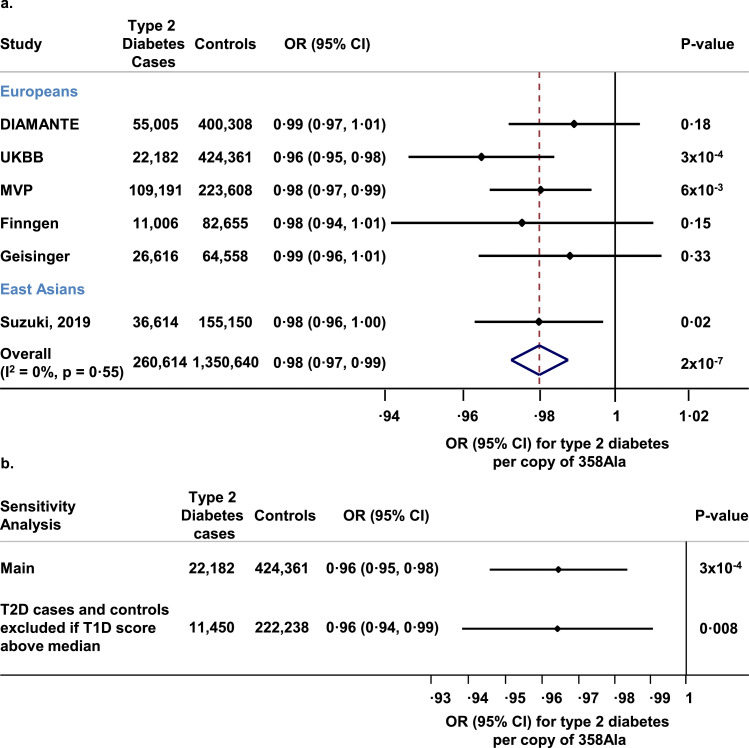
Association of *IL6R* Asp358Ala with type 2 diabetes, glycaemic and anthropometric traits. Panel a. Association between *IL6R* Asp358Ala and type 2 diabetes. Odds ratios and their 95% confidence intervals for type 2 diabetes are presented per copy of 358Ala. **Panel b.** Association of Asp358Ala with type 2 diabetes in UK Biobank before and after excluding participants based on the values of a type 1 diabetes polygenic risk score. Both cases and controls were excluded if the type 1 diabetes score was above the median of the value among diabetes cases. Abbreviations: DIAMANTE, Diabetes meta-analysis of trans-ethnic association studies; UKBB, United Kingdom biobank; MVP, Million veteran program OR, odds ratio; CI, confidence interval; Asp, aspartic acid; Ala, alanine; T2D, type 2 diabetes; T1D, type 1 diabetes.

In 14,528 participants from the Fenland and EPIC—Norfolk cohorts, each copy of the 358Ala partial loss-of-function variant in *IL6R* was associated with 0·11 log-pg/mL (95% CI, 0·09, 0·13; *P*<·001; linear regression) higher IL-6 levels. In 260,614 cases and 1350,640 controls, each copy of the 358Ala partial loss-of-function variant in *IL6R* was associated with an odds ratio for T2D of 0·98 (95% CI, 0·97, 0·99; *P*<·001; logistic regression; Fig. 3a). In UK Biobank, the odds of T2D was lowest in carriers of two copies of the 358Ala allele (OR compared with non-carriers, 0·92; 95% CI, 0·89, 0·96; *P*<·001; logistic regression; eFigure 5), with no statistical evidence of deviation from an additive association (P_non-linearity_=0·13; logistic regression; eFigure 5). In the UK Biobank study, estimates of association with T2D for Asp358Ala were comparable in odds ratio, variance explained and heritability to those for coronary artery disease (eFigure 5 and eTable 9). The association of Asp358Ala with diabetes was conditionally independent of an association peak for T2D led by rs2481065, which is located 115,059 base pairs upstream of the Asp358Ala variant (OR per copy of 358Ala conditioned on rs2481065, 0·97; 95% CI, 0·95, 0·99; *P* = 007; logistic regression; eTable 10). The association with T2D of the Asp358Ala variant was not the consequence of case-admixture (diagnostic misclassification) between type 1 and T2D. First, in an analysis including 24,209 cases and 758,240 controls, each copy of the 358Ala allele was associated with an odds ratio for type 1 diabetes of 0·97 (95% CI, 0·95, 0·99; *P*=·007; logistic regression; eFigure 6). Given the ~10-fold higher prevalence of type 2 vs type 1 diabetes in the general population [53] and similar magnitude of association, only very high levels of misclassification might lead to a spurious association with T2D (eFigure 7). To further address this, a sensitivity analysis was conducted in UK Biobank where possible cases of type 1 diabetes were excluded on the basis of a cut-off of a polygenic score for type 1 diabetes (sensitivity to detect type 1 diabetes cases for exclusion >95%) [53]. In this analysis, estimates of the association with T2D were approximately the same as those in the main analysis (OR for T2D, 0·96; 95% CI, 0·94, 0·99; *P* = 008; logistic regression; Fig. 3b).

In continuous metabolic trait analyses, the 358Ala allele was associated with lower HbA1c (beta in SD units per copy of 358Ala, −0·007; 95% CI, −0·012, −0·003; *P* = 002; linear regression; eFigure 8) and numerically lower BMI-adjusted WHR (beta in SD units per copy of 358Ala, −0·004; 95% CI, −0·008, 0; *P* = 03; linear regression; eFigure 8). The latter association was not statistically significant after accounting for the number of tests and was unlikely to fully explain the association with T2D (eFigure 8).

The association of Asp358Ala genotype was similar in people above or below the median of 10 polygenic scores for T2D risk factors, with no evidence of interaction (eFigure 9a). There was, however, evidence of a significant interaction between Asp358Ala genotype and a polygenic score for higher BMI on HbA1c levels (P_interaction_=0·009; linear regression; eFigure 9b). Projections of the range of possible effects of IL-6R inhibition therapy in the primary prevention of T2D were generated based on results from genetic studies and biomarker data from trials. These projections suggested that treatment might attain benefits above 10% relative risk reduction, corresponding to a relative risk of 0·90, only when assuming long-term treatment duration with high dose inhibitors (eFigure 10).

### 4.3 Mediation by the IL-6 pathway of cardio-metabolic disease risk associated with higher adiposity

Both higher measured BMI or genetic predisposition to higher BMI via a 97-variant polygenic score were associated with higher circulating IL-6 levels (0·14 log-pg/mL higher IL-6 levels per 4·8 kg/m^2^ [corresponding to one standard deviation] higher measured BMI; 95% CI, 0·12, 0·16; *P*<·001; linear regression; 0·13 log-pg/mL higher IL-6 levels per 4·8 kg/m^2^ higher BMI due to the polygenic score; 95% CI, 0·05, 0·21; *P*=·001; linear regression). The percentage of the BMI/T2D association mediated by IL-6 was estimated to be 4% (95% CI, 3, 5%) in observational analyses and 3% (95% CI, 2, 5%) in genetic analyses. The percentage of the BMI/cardio-metabolic disease association mediated by IL-6 was estimated to be 18% (95% CI, 14, 25%) in observational analyses and 10% (95% CI, 7, 15%) in genetic analyses. The percentage of the association between BMI and fasting insulin levels mediated by IL-6 was estimated to be 3% (95% CI, 1, 6%) in genetic analyses.

## 5 Discussion

This large genetic and biomarker study supports the hypothesis that IL-6 mediated inflammation plays a role in the etiology of T2D, but it also suggests that the impact of this pathway on disease risk in the general population may be small. Early evidence of a possible role of inflammation in metabolic disease dates back to over 25 years ago [3]. Findings of an inflammatory response accompanying obesity [5,9,61,62], insulin resistance [5,9,13] and diabetes [9,14,61,62], and the support of experimental results from cellular and animal models have led to the hypothesis that T2D may be viewed as an “inflammatory disease” [1].

This study provides large-scale human genetics evidence that supports the role of chronic inflammation via the IL-6 pathway in T2D. It also shows that IL-6 mediates in small part the links between obesity, insulin resistance and cardio-metabolic diseases, which has been a prevalent hypothesis in the field due to the evidence that the adipose tissue of insulin-resistant obese people is infiltrated with inflammatory cells [5,63] and displays an enhanced secretion of IL-6, tumor necrosis factor alpha and other pro-inflammatory mediators [13,61,62]. Biomarker [8,[21], [22], [23]] and human genetic [24], [25], [26] results with similar strength to those of this study have formed part of the evidence basis for efforts to re-purpose IL-6R inhibitory therapy in cardiovascular disease (e.g. clinicaltrials.gov registered trials number NCT03004703, NCT01491074, or NCT02419937).

However, several findings from this study suggest that the impact of the IL-6 pathway on risk of T2D in the general population may be small. Firstly, people in the top decile of IL-6 levels had a 2-fold (hazard ratio, 2·14) higher risk of incident T2D than people in the bottom decile. This is approximately three times smaller than the relative risk associated with being in the top decile of BMI in our study (hazard ratio, 5·64). While the association between Asp358Ala and lower T2D risk was statistically robust, consistent across studies and ancestries and was unaffected by possible misclassification of type 1 diabetes cases, the odds ratio of 0·98 for disease risk was lower for carriers of one copy and the odds ratio of 0·92 was lower for carriers of two copies of this partial loss-of-function allele compared to non-carriers. Finally, the proportion of BMI-associated risk of diabetes mediated by IL-6 levels was below 5%, ~3–4 times smaller than for coronary disease. In line with this, the proportion of the association between BMI and fasting insulin levels mediated by IL-6 was around 3%. Taken together, these results suggest that removing inflammation via this pathway may not substantially impact on diabetic risk in obesity but still represents a biologically significant effect.

This study has limitations. First, as this study is based on observational epidemiology findings and not randomised controlled trial data, it cannot establish causality. Second, this study did not exclude patients with pre-existing inflammatory conditions or acute infections at baseline. Although we cannot exclude the possibility that IL-6 levels may have been impacted by acute infections at baseline, to affect the observational results presented, IL-6 levels would have to differ significantly at the time of prospective type 2 diabetes diagnosis to have an effect. Coupled with this, the study design included follow-up of these observational analyses with genetic analyses which are robust to this type of confounding. Despite these efforts to mitigate this potential confounding, capturing infection-mediated inflammation in observational studies is challenging and is therefore a limitation of this study. Third, some of the findings of this study assume that the *IL6R* Asp358Ala variant mimics the effects of IL-6R inhibition. While the *in vitro* consequences [27] and the pattern of association [24,25] of this variant with a variety of phenotypes have been shown to be consistent with the effects of IL-6R inhibitory drugs, differences cannot be excluded which could affect some of the estimates reported here. Using re-scaling methods to project the possible efficacy of IL-6R blocking therapy in primary prevention based on estimates of lifelong genetic associations, the projected relative risk reduction was below 10% in most simulated scenarios. In comparison, relatively safe and inexpensive lifestyle interventions [64], metformin [64] or other hypoglycaemic drugs [65] have been associated with reductions in the incidence of diabetes ranging from 30% to 70% in previous primary prevention trials. However, due to the underlying assumptions, this estimate is likely to be conservative and may underestimate the effect of IL-6R antagonists, which profoundly inhibit the pathway. In contrast to pharmacological antagonism, the *IL6R* Asp358Ala variant results in a partial loss of IL-6 mediated signaling and may therefore not be the most informative model to estimate the possible effects IL-6R blockade on glucose metabolism achieved through complete or near-complete pharmacological inhibition of this pathway. Coupled with this, IL-6 is a pleiotropic cytokine that has established pro- and anti-inflammatory effects that act via the classical and trans signaling pathways [66]. It is therefore conceivable that the Asp358Ala variant acts upon the anti-inflammatory effects of IL-6 signaling and may have impacted upon the results of this study. Fourth, while some of the results from this study suggest that IL-6R inhibition might have limited efficacy for the primary prevention of T2D, these findings focus on first occurrence of disease in people from the general population. Hence, these findings do not exclude that IL-6R inhibition may yield clinically significant benefits on glucose metabolism in groups of people at risk for T2D, or on glycaemic levels and risk of complications in patients with T2D. In fact, in a post-hoc meta-analysis of three randomized controlled trials of the IL-6R inhibitor sarilumab including 1982 patients with rheumatoid arthritis, Genovese and colleagues showed statistically-significant improvements in HbA1c in patients randomized to sarilumab as opposed to placebo (least squares mean difference in HbA1c ranging from −0·21% to −0·69%), which were more pronounced at higher dosages and in patients who also had diabetes [67]. This finding is further reinforced by results demonstrating a significant 0·4% reduction in HbA1c in 67 rheumatoid arthritis patients treated with tocilizumab [68]. Therefore, it is possible that IL-6R inhibition may yield clinically meaningful changes in glycaemia in people with inflammatory or immune conditions which are linked with diabetes. Results from our interaction analysis between Asp358Ala and BMI on HbA1c levels suggest that reductions in HbA1c may be more pronounced in individuals below the median of the BMI polygenic score. However, as this result was estimated in a population-based cohort, this requires validation in a cohort selected for participants with existing inflammatory conditions. Finally, while the associations observed in this study are statistically robust and despite a growing body of research in the topic, the molecular mechanisms linking the IL-6 inflammatory pathway with glucose metabolism remain only partly understood. In this study, IL-6 levels and the *IL6R* Asp358Ala variant were more strongly associated with HbA1c, which reflects average glycaemic levels in the three months prior to measurement, than with other glycaemic traits. Infection and other inflammatory challenges are associated with reactive hyperglycaemia [69], which may be aggravated in people with higher IL-6 levels [70]. It is possible that enhanced glycaemic response to inflammatory stimuli may explain part of the associations between higher IL-6 mediated inflammation and higher diabetes risk observed in this study. This large human genetics and biomarker study supports the hypothesis that IL-6 mediated inflammation is implicated in the pathophysiology of T2D but suggests that the impact of this pathway on disease risk is likely to be small.

## Contributors

Nicholas Bowker designed the study, analysed the data and wrote the first draft of the report. Claudia Langenberg, Nicholas J. Wareham and Luca A. Lotta supervised the study. Nicholas Bowker and Rupal L. Shah performed the literature search and reviewed the identified articles. Stephen J. Sharp provided statistical support. Nicholas Bowker, Luca A. Lotta, Jian'an Luan, Isobel D. Stewart, and Eleanor Wheeler collated the data and contributed to the data analysis. Manuel A. R. Ferreira and Aris Baras provided data from the MyCode Study from the DiscovEHR Collaboration between Regeneron Genetics Center and Geisinger Health System and are designated members of the writing team for Regeneron Genetics Center and the DiscovEHR Collaboration. All authors contributed to the interpretation of results and writing or revision of the manuscript.

## Declaration of Competing Interest

M.A.R.F., A.B., L.A.L. are employees and shareholders of Regeneron Pharmaceuticals. N.B., R.L.S., S.J.S., J.L., I.D.S, E.W., N.J.W. and C.L. have nothing to declare.

## Appendix Supplementary materials

Image, application 1

Image, application 2

Image, application 3

## Footnotes

Supplementary material associated with this article can be found in the online version at doi:10.1016/j.ebiom.2020.103062.
