# Supplementary material for: Meta-analysis investigating the role of interleukin-6 mediated inflammation in type 2 diabetes
Source: eBioMedicine. 2020 Oct 21;61:103062. doi: 10.1016/j.ebiom.2020.103062 (PMC7581887; doi:10.1016/j.ebiom.2020.103062)
Supplement: Supplementary file 1 [file mmc1.docx]

# **Online Supplement**

Meta-analysis investigating the role of interleukin-6 mediated inflammation in type 2 diabetes

Index

eMethods – Page 2

eTables – Page 5

eFigures – Page 24

eReferences – Page 35

**eMethods 1. Additional methods for observational epidemiology analyses.**

*Systematic review and meta-analysis of prospective studies investigating the association of IL-6 levels with incident type 2 diabetes*

A systematic review and meta-analysis of studies investigating the association of IL-6 levels with incident type 2 diabetes in population-based cohorts was conducted. A previous meta-analysis had been performed by Wang et al^1^ for studies with a publication date up to 10 February 2012. In addition to studies identified by Wang et al^1^, novel results from the EPIC-Norfolk study and from studies identified with a new and updated systematic search were included in the meta-analysis reported in this manuscript. A review protocol has not previously been published and will be described in detail in this section. EPIC-Norfolk constitutes the largest study to date and the one with the longest duration of follow-up (**eTable 2**). A search of the PubMed electronic database was performed to identify studies investigating the association of IL-6 levels with incident type 2 diabetes. Prospective studies published between 10 February 2012 and 30 October 2018 were considered. A full list of search terms can be found in **eTable 3**. Studies focusing on prevalent type 2 diabetes, type 1 diabetes or gestational diabetes were excluded as were review articles and animal studies. Additionally, studies were excluded if they included cohorts that overlapped those already included in the meta-analysis by Wang and colleagues. The initial database search yielded 379 recent publications, the titles of which were initially screened using the above-mentioned criteria. Two of the authors (NB and RLS) independently screened the titles of the identified publications and conferred to ensure consistency. Following this, 14 relevant publications were identified, and their abstracts were further screened by the same authors to ensure consistency. Overall, three recently published studies^2–4^ were identified and their results were included in the meta-analysis, which included 15 studies overall. Quality ratings for the 15 selected studies were adjudicated based on criteria evaluating study size and representativeness, reliability of exposure measurement, reliability of type 2 diabetes ascertainment, and adjustment for possible confounders (**eTable 4**).

Estimates of association (risk ratios, odds ratios, hazard ratios – all assumed to approximate the hazard ratio) with type 2 diabetes were calculated per 1 log pg/mL higher IL-6 levels for studies included in the Wang *et al*^1^ analysis. In the case of the three newly identified studies, only the results of Koloverou *et al*^4^ were presented per 1 log pg/mL higher IL-6 levels. The results of Dallmeier *et al*^2^ and Marques-Vidal *et al*^3^ were originally presented per 1 SD higher log IL-6 levels. These estimates were converted to units per 1 log pg/mL higher IL-6 levels using a conversion factor of 0·8 (corresponding to the SD of log IL-6 levels in the EPIC-Norfolk study). Study characteristics and relative risks for the included studies are summarised in **eTable 2**. Fixed-effects meta-analysis was conducted including all 15 available studies consisting of a total of 5,421 incident type 2 diabetes cases and 31,562 non-cases. The combined hazard ratio was reported per 1 log pg/mL higher IL-6 levels. The I^2^ heterogeneity statistic was used to estimate significant heterogeneity between study effect estimates.

To assess potential publication bias, the Egger test^5^ was used. Furthermore, funnel plots illustrating the relationship between the hazard ratio and standard error of each study were drawn (**eFigure 11**). No significant evidence of publication bias was found (P = 0·44). The funnel plot showed that two studies were on the contour line and could potentially bias the overall estimate (**eFigure 11**). Subgroup analysis excluding these two studies demonstrated no change in the overall estimate (1·24; 95% CI, 1·17, 1·32; P=1x10^-12^). We also sought to estimate potential bias induced via differing IL-6 measurement methods between studies. Three studies that measured IL-6 levels using techniques other than electrochemiluminescent methods such as ELISA were excluded. The remaining prospective studies were meta-analysed, resulting in the same effect estimate as the main analysis (HR 1·24; 95% CI, 1·16, 1·33; P = 1x10^-10^). Differential IL-6 measurements between studies were therefore ruled out as a potential source of bias.

*DEXA protocol in Fenland.*

In the Fenland study overall and regional body fat mass was quantified by dual-energy X-ray absorptiometry (DEXA) using a Lunar Prodigy advanced fan beam scanner (GE Healthcare, Bedford, UK), encore v14·10·022 and CoreScan® software (GE Healthcare, Bedford UK). All body images were manually processed, and demarcations corrected following a standardized protocol. The trunk included neck, chest, abdominal and pelvic areas. The legs were defined as the region below the lower borders of the trunk. The abdomen was defined as the portion of the trunk between ribs and pelvis, defined by the outline of the iliac crest. The gluteofemoral region included hips and upper thighs. Visceral abdominal fat mass was estimated using subcutaneous fat width and the anteroposterior thickness of the abdominal wall. These measurements were used to extrapolate abdominal subcutaneous fat mass. Visceral abdominal fat mass was calculated by subtracting the subcutaneous abdominal fat mass from the total abdominal fat mass.

**eMethods 2. Additional methods for genetic association analyses.**

*Re-scaling the association estimates of IL6R Asp358Ala with continuous metabolic traits*

To aid the interpretation of association estimates with different continuous metabolic traits (e.g. HbA1c or fasting insulin), the associations were reported in both clinical and standardized units of each trait per copy of the *IL6R* 358Ala allele. Clinical units aid interpretation of the association results, while standard deviation units enable comparison of association estimates across outcomes. Estimates from summary-level results were converted to standardized units or to clinical units using the standard deviation (SD) in the Fenland or in the UK Biobank as summarized in **eTable 6**.

In these analyses, individuals carrying the *IL6R* 358Ala allele had numerically lower WHR adjusted for BMI, a risk factor for type 2 diabetes^6^ However, the difference in WHR adjusted for BMI observed for the *IL6R* 358Ala allele is unlikely to explain its association with lower risk of type 2 diabetes. From previous analyses, a 1-SD increase in WHR adjusted for BMI is associated with an odds ratio for type 2 diabetes of 1·79^6^ On the basis of this estimate, given that each copy of *IL6R* 358Ala is associated with 0·004 SD lower BMI-adjusted WHR, the expected odds ratio for type 2 diabetes would be 0·998 (i.e. a very small estimated effect size) if the association of the *IL6R* 358Ala allele was entirely due to BMI-adjusted WHR. Since the 95% confidence interval estimated for the association between the *IL6R* 358Ala allele and type 2 diabetes excludes 0·998 (i.e. odds ratio per allele from 0·970 to 0·989), it is unlikely that the association of this variant with diabetes is only due to an association with more favourable fat distribution.

*Estimation of heritability and variance in the risk for type 2 diabetes and coronary disease explained by the IL6R 358Ala allele*

In stage 2, estimates of heritability for type 2 diabetes and coronary artery disease were obtained using linkage disequilibrium score regression^7^ in UK Biobank using the HapMap3 European ancestry CEU population as reference panel (**eTable 9**). Variants included in the regression model were restricted to those present in the reference panel with minor allele frequency above 5% to retain only common variants. Type 2 diabetes and coronary artery disease prevalence in the general population were assumed to be equivalent to the prevalence in UK Biobank, 5·51% and 5·50% respectively. The heritability in these conditions explained by the *IL6R* 358Ala allele was estimated by dividing the heritability of the disease by the variance explained by the genetic variant.

*Exclusion of misdiagnosed cases of type 1 diabetes using a polygenic score approach*

To assess whether the association with type 2 diabetes of *IL6R* 358Ala might be affected by misclassification of cases of type 1 diabetes as type 2 diabetes, an approach based on a 29-variant polygenic risk score^8^ for type 1 diabetes was used. Using individual-level data from UK Biobank, this polygenic score was calculated for each participants by adding the number of copies of each contributing variant weighted by its per allele association estimate with type 1 diabetes as previously described^8^.

Individuals with values of the polygenic score below the median in people with diabetes (type 1 and type 2 combined; value in UK Biobank, 13·04) have been shown to be highly unlikely to have type 1 diabetes (sensitivity to detect type 1 diabetes cases for exclusion, 96%; false negative rate, 4%)^8^ This means that if one were to restrict an analysis to people below that value, only 4% of type 1 diabetes cases originally included would remain in the analysis. This restriction does not bias the association of the *IL6R* 358Ala allele as the *IL6R* locus is not included in the type 1 diabetes genetic score and therefore is in linkage equilibrium with (i.e. inherited randomly regarding) the loci included in the score.

Given that type 1 diabetes accounts for ~10% of diabetes in the general population, one would expect that amongst the approximately 22,000 cases of type 2 diabetes included in this analysis, cases of type 1 diabetes erroneously classified as type 2 diabetes would be 2,200 if there were a 100% misclassification. Even in the extreme scenario of 100% misclassification, restricting to only people with polygenic score for type 1 diabetes below the median would lower the number of type 1 diabetes present in the analysis to just 44 (0·4% of overall diabetes cases; corresponding to 1,100 misclassified cases left in the halved sample size multiplied by 4% false negatives). More likely scenarios of a lower misclassification rate yield numbers of misclassified type 1 diabetes cases left in the sensitivity analysis in the single digits (**eFigures 7B and 7C**). On this basis, the association with type 2 diabetes was estimated after restricting for participants below the median of the type 1 diabetes polygenic score.

*Projection of the potential benefit of IL6R blocking therapy on the risk of type 2 diabetes.*

Both experimental^9^ and genetic^10,11^ association studies have shown that rs2228145-C (358Ala) closely mimics some of the effects of IL-6R inhibitory therapy. However, an association of this variant with lower risk of type 2 diabetes, as shown in this study, does not necessarily mean that IL-6R inhibitory therapy will produce clinically meaningful reduction in the risk of type 2 diabetes. Even if the effects of a genetic variant and that of a drug on the target were qualitatively the same, two differences between genetic variant and treatment with the drug in randomized clinical trials would remain. First, genetic variants are usually associated with small differences in the activity of the target gene, while drugs usually have large effects on target activity (different effect size)^12,13^. Second, differences in target gene activity associated with a genetic variant are lifelong, while the effect of drugs is usually assessed in trials of short duration (duration of exposure). With several assumptions, these differences can sometimes be modelled to formulate projections of the likely efficacy of drug treatment based on association magnitude of genetic variants that mimic that drug treatment.

In this study, projections of potential benefit of IL-6R blocking therapy on the risk of type 2 diabetes in a primary prevention setting were formulated using (a) the genetic association of *IL6R* 358Ala with type 2 diabetes from this study, (b) the genetic association of *IL6R* 358Ala with C-reactive protein (CRP; used as biomarker of target engagement for IL-6R) in this and other studies^10^ and (c) the effects of IL-6R blocking therapy on CRP (the most-widely used biomarker of target engagement for IL-6R blocking therapy) in randomized clinical trials of tocilizumab, an IL-6R inhibitor^11^.

First, to model the different effect size, ratios between the absolute differences in CRP levels for the Asp358Ala variant and for IL-6R inhibitory therapy were obtained. Estimates of the absolute difference in CRP between IL-6R inhibitory therapy (tocilizumab 4 or 8 mg/kg) and placebo was obtained from a published meta-analysis^11^ of randomized controlled trials. Estimates of the absolute difference in CRP per copy of the 358Ala allele were obtained on the basis of previously-published genetic association studies^10^. Because CRP was log_e_-transformed in these studies, the reported estimate reflected the percentage rather than the absolute difference (i.e., 7·5% lower CRP per allele). To obtain an absolute difference comparable to the one reported in the trials (which were conducted in people with autoimmune disease and hence high average CRP levels), the 7·5% difference was applied to the mean CRP level of individuals contributing to the trial estimate (i.e., mean CRP in trial participants, 26 mg/L; absolute difference per copy of 358Ala, -1·9 mg/L). This assumes that the percentage difference in CRP due to the 358Ala allele stays the same at different starting levels of CRP. To assess this, conditional quantile regression (CQR) and subsequent meta-regression were performed in 14,695 individuals from EPIC-Norfolk. Using the CQR model, the association of 358Ala with ln-CRP was estimated at every 5^th^ percentile of the ln-CRP distribution. Standard errors were calculated using bootstrapping with 200 replicates. CQR models were adjusted for age, sex and the first four genetic principal components. Meta-regression across the CQR estimates per quantile was performed to estimate the difference in ln-CRP per copy of 358Ala across the quantiles of ln-CRP. The meta-regression model was adjusted using the same covariates as the CQR. This meta-regression model did not show evidence that the variant association estimates vary at different ln-CRP levels (P=0·81; **eFigure 2**). Therefore, ratios between the absolute differences in CRP levels for the 358Ala allele and IL-6R inhibitory therapy were estimated (i.e. 4·7 for tocilizumab 4 mg/kg / 358Ala and 10·9 for tocilizumab 8 mg/kg / 358Ala). These ratios were used to re-scale the estimate of association with type 2 diabetes from genetic association studies of Asp358Ala while accounting for the larger absolute effect of IL-6R inhibitory therapy on CRP. This re-scaling makes the critical assumption that the effects of IL-6R inhibition on diabetes risk estimated by a small difference due to the genetic variant will scale linearly even for large (~5-10 fold) differences between variant and treatment effects.

This estimate attempts to correct for the different effect size of genetic variant vs drug but still does not correct for the duration of exposure. To account for that, it was assumed that the rescaled estimate represented the projected effect of the drug on diabetes risk for a lifelong-exposure (or a long-term exposure of several decades as genetic association studies used for these estimates were conducted in people with an average age of ~59 years). Projections for shorter exposure were obtained by re-scaling the life-long estimate for increasing numbers representing the ratio of exposure between lifelong and a given shorter exposure. Estimates are reported for ratios ranging from 1 to 10, to capture a range of hypothetical durations of treatment. This projection assumes a constant additive risk of the IL-6 pathway on type 2 diabetes risk, which is in line with assumptions of genetic association studies of common variants and previously used Mendelian randomization approaches in this and other settings. These projections are relative to primary occurrence of type 2 diabetes in a general population setting and do not reflect the likely efficacy of treatment in people with high diabetes risk or with inflammatory conditions (e.g. people with rheumatoid arthritis).

**eTable 1. Summary of the studies participating in the different analyses of the manuscript.**

| **Study stage ^a^** | **Outcome** | **Cases overall, N** | **Non-cases (for case-control studies) or participants (for continuous trait studies) overall, N** | **Participating study** | **PubMed ID for cohort description** | **Website (URL)** |
| --- | --- | --- | --- | --- | --- | --- |
| 1 | Type 2 diabetes | 5,421 | 31,562 | 15 prospective studies | 10466767;  23264288;  23130155;  23251619;  28834086 | http://www·srl·cam·ac·uk/epic/ |
|  | 2-hour glucose | - | 10,218 | Fenland | 27841877 | http://www·mrc-epid·cam·ac·uk/research/studies/fenland/ |
|  | Fasting glucose | - | 10,344 |  |  |  |
|  | HbA1c | - | 10,331 |  |  |  |
|  | Fasting insulin | - | 8,428 |  |  |  |
|  | DEXA traits | - | 10,344 |  |  |  |
|  | Iron | - | 8,980 |  |  |  |
|  | Transferrin | - | 8,988 |  |  |  |
|  | Ferritin | - | 8,958 |  |  |  |
| 2 | Type 2 diabetes | 22,182 | 424,361 | UK Biobank | 25826379 | http://www·ukbiobank·ac·uk/ |
|  |  | 109,191 | 223,608 | Million Veteran Program | 26441289 | https://www·research·va·gov/mvp/ |
|  |  | 55,005 | 400,308 | DIAMANTE | 30297969 | N/A |
|  |  | 36,614 | 155,150 | Suzuki *et al·* | 30718926 | http://jenger·riken·jp/en/result |
|  |  | 11,006 | 82,655 | Finngen | NA | https://www·finngen·fi/en |
|  |  | 26,616 | 64,558 | Geisinger | 26866580 | https://www·geisinger·org/mycode |
|  | Type 1 diabetes | 12,079 | 12,262 | T1DGC | 17130525 | https://repository·niddk·nih·gov/studies/t1dgc/ |
|  |  | 11,182 | 321,617 | Million Veteran Program | 26441289 | https://www·research·va·gov/mvp/ |
|  |  | 948 | 424,361 | UK Biobank | 25826379 | http://www·ukbiobank·ac·uk/ |
|  | Coronary artery disease | 24,890 | 427,309 |  |  |  |
|  | Fasting glucose | - | 133,010 | MAGIC | 22885924; 22581228 | https://www·magicinvestigators·org/ |
|  | 2-hour glucose | - | 42,854 |  |  |  |
|  | Fasting insulin | - | 108,557 |  |  |  |
|  | HbA1c | - | 479,942 | MAGIC; UK Biobank | 28898252; 25826379 | https://www·magicinvestigators·org/; http://www·ukbiobank·ac·uk |
|  | Non-fasted glucose | - | 355,817 | UK Biobank | 25826379 | http://www·ukbiobank·ac·uk |
|  | Body mass index | - | 772,066 | GIANT; UK Biobank | 25673413;  25826379 | https://portals·broadinstitute·org/collaboration/  giant/index.php/GIANT_consortium;  http://www·ukbiobank·ac·uk |
|  | Hip circumference | - | 604,143 |  |  |  |
|  | Waist circumference | - | 615,305 |  |  |  |
|  | Waist-to-hip ratio adjusted for BMI | - | 625,123 |  |  |  |
|  | Waist-to-hip ratio unadjusted | - | 602,940 |  |  |  |
| 3 ^b^ | Type 2 diabetes (observational association) | 411 | 7,010 | EPIC-Norfolk | 10466767 | http://www·srl·cam·ac·uk/epic/ |
|  | Coronary artery disease  (observational association) | 1,123 | 5,917 |  |  |  |
|  | Type 2 diabetes  (genetic association) | 17,006 | 330,572 | UK Biobank | 25826379 | http://www·ukbiobank·ac·uk/ |
|  | Coronary artery disease  (genetic association) | 18,770 | 333,247 |  |  |  |

Abbreviations: N, Number of participants; HbA1c; Glycated haemoglobin; BMI; Body mass index; EPIC, European prospective investigation of cancer; DIAMANTE, Diabetes meta-analysis of trans-ethnic association studies; MAGIC, Meta-Analyses of glucose and insulin-related traits consortium; T1DGC, Type 1 diabetes genetics consortium.

1. Traits are continuous unless otherwise stated as a disease or a polygenic score
2. EPIC-Norfolk contributed to both the observational and genetic mediation analyses whereas UK Biobank contributed to only the genetic mediation analysis

**eTable 2. Study characteristics and adjusted risk ratios from studies of incident type 2 diabetes associated with levels of IL-6.**

| **Study, year (country)** | **Cases, N** | **Non-cases, N** | **Proportion of women (%)** | **Study design** | **Duration**  **(years of follow-up)** | **Type 2 diabetes ascertainment** | **Exposure measurement method** | **Risk Ratio**  **(95% CI)** | **Covariates** | **Source** |
| --- | --- | --- | --- | --- | --- | --- | --- | --- | --- | --- |
| Pradhan et al. 2001 (US) | 188 | 362 | 100 | Nested case-control | 4 | Self-report based on ADA criteria and verified by primary care records | ELISA (R&D Systems, Minneapolis, MN) | 1·70  (0·61-4·74) | Age, FH of diabetes, smoking, physical activity, alcohol, PMT, fasting status, BMI, and FI | a |
| Duncan et al. 2003 (US) | 581 | 572 | 60 | Case cohort | 9 | One of:  1. Reported physician diagnosis;  2. use of anti-diabetes medications;  3. FG ≥7·0 mmol/L;  4. non-fasting glucose of ≥11·1 mmol/L | ELISA (R&D Systems, Minneapolis, MN) | 1·59  (1·02-2·48) | Age, centre, sex, ethnicity, FH of diabetes, hypertension, BMI, WHR, FG, and FI | a |
| Krakoff et al. 2003 (US) | 71 | 71 | 66 | Nested case-control | 4·6 | WHO criteria | ELISA (Quantikine High Sensitivity; R&D Systems, Oxon, U·K·) | 0·75  (0·45-1·25) | Age, WC, FPG, PG2h, HbA1c, and FI | a |
| Spranger et al. 2003 (Germany) | 188 | 377 | 21 | Nested case-control | 2·3 | Self-report with cases validated using a questionnaire completed by the primary care physician | ELISA (R&D Systems, Minneapolis, MN) | 2·20  (1·31-3·69) | Age, sex, sporting activities, smoking, alcohol, educational attainment, BMI, WHR, and HbA1c | a |
| Hu et al. 2004 (US) | 737 | 785 | 100 | Nested case-control | 10 | Unconfirmed self-report of one of:  1. Classic symptoms plus elevated glucose levels;  2. two elevated plasma glucose if no symptoms;  3. treatment with agents or insulin, ADA criteria for cases after 1998 | ELISA (Quantikine HS Immunoassay kit) | 1·52  (1·18-1·96) | Age, race, time at blood drawn, alcohol, physical activity, smoking, FH of diabetes, PMT, MP, diet score, fasting status, and BMI | a |
| Liu et al. 2007 (US) | 1584 | 4317 | 100 | Nested case-control | 5·9 | Self-report | ELISA (R&D Systems) | 1·19  (1·02-1·39) | Age, race, clinic, time of blood draw, BMI, alcohol, physical activity, smoking, PMT, FH of diabetes, and FG | a |
| Thorand et al. 2007 F (Germany) | 222 | 809 | 100 | Case cohort | 10·8 | Self-report with cases validated using a questionnaire mailed to the treating physician or by medical chart review | High-sensitivity latex enhanced nephelometric assay on a BN II analyser (Dade-Behring, Marburg, Germany) | 1·65  (1·16-2·35) | Age, survey, smoking, alcohol, physical activity, SBP, total-to-HDL cholesterol ratio, BMI, and FH of diabetes | a |
| Thorand et al. 2007 M (Germany) | 305 | 889 | 0 | Case cohort | 10·8 | Self-report with cases validated using a questionnaire mailed to the treating physician or by medical chart review | High sensitivity immunoradiometric assay (IRMA) (men aged 45–64 years) or a high-sensitivity latex enhanced nephelometric assay on a BN II analyser (men aged 35–44 years) (Dade-Behring, Marburg, Germany) | 1·34  (1·06-1·69) | Age, survey, smoking, alcohol, physical activity, SBP, total-to-HDL cholesterol ratio, BMI, and FH of diabetes | a |
| Wannamethee et al. 2007 (UK) | 108 | 3599 | 0 | Cohort | 5 | Self-report confirmed via primary care records | ELISA (R&D Systems) | 1·26  (1·05-1·51) | Age, social class, physical activity, smoking, alcohol, use of statins, CHD or stroke, SBP, treatment for hypertension, BMI, and HOMA-IR | a |
| Ley et al. 2008 (Canada) | 86 | 406 | 58 | Cohort | 10 | One of:  1. FG ≥7·0 mmol/L or PG2h ≥11·1 mmol/L;  2. current use of insulin or oral hypoglycaemic agents;  3. self-report (not verified from records) | ELISA (BioSource International, Camarillo, CA) | 0·98  (0·74-1·30) | Age, sex, TG, HDL-C, hypertension, WC, and IGT | a |
| Bertoni et al. 2010 (US) | 410 | 5161 | 53 | Cohort | 4·7 | Using hypoglycaemic medication or FG ≥7 mmol/L | ELISA (Quantikine HS Human IL-6 Immunoassay; R&D Systems) | 1·30  (1·09-1·55) | Age, sex, race, education, site, alcohol, smoking, exercise, SBP, antihypertensive medication, HOMA-IR, and BMI | a |
| Dallmeier et al·, 2012 (USA) | 162 | 2476 | 56 | Cohort | 6·6 | Fasting glucose level ≥126 mg/dL or use of insulin or oral hypoglycaemic medications at follow-up | ELISA (R&D Systems, Minneapolis, MN) | 1·09  (0·84-1·40) | Age, sex, cohort, BMI, FG, SBP, HDL-C, TG, and smoking | b |
| Marques-Vidal et al·, 2012 (Switzerland) | 208 | 3634 | 56·7 | Cohort | 5·5 | Fasting plasma glucose ≥7·0 mmol/L and/or presence of oral hypoglycaemic or insulin treatment | Multiplexed particle-based flow cytometric cytokine assay | 1·06  (0·85-1·32) | Age, FH of type 2 diabetes, height, WC, resting heart rate, presence of hypertension, HDL-C, TG, FG and serum uric acid | b |
| Koloverou et al·, 2018 (Greece) | 160 | 1094 | 51 | Cohort | 10 | Unconfirmed self-report or ADA criteria i·e. use of antidiabetic medication (hypoglycaemic drugs and/or insulin use) or fasting plasma glucose ≥126 mg/dL | ELISA | 1·08  (0·19-6·09) | Age, sex, FH of diabetes, smoking, adherence to the Mediterranean diet, physical activity, hypertension, hypercholesterolaemia status and BMI | b |
| EPIC-Norfolk, (UK) | 411 | 7010 | 61 | Cohort | 16·7 | 1. WHO criteria (HbA1c >6·5% (48mmol/mol))  2. Electronic health records (ICD 10: E11)  3. Self-report | Electrochemiluminescence (Meso Scale Discovery, US) | 1·25  (1·10-1·41) | Age, sex, BMI, WHR, ethnicity, education level, FH of type 2 diabetes, smoking status, average units of alcohol per week and average physical activity per week | c |

Abbreviations: ADA, American Diabetes Association; BMI, body mass index; CHD, coronary heart disease; FG, fasting glucose; FH, family history; FI, fasting insulin; FPG, fasting plasma glucose; HDL-C, HDL cholesterol; IGT, impaired glucose tolerance; MP, menopausal; PG2h, 2-h plasma glucose; PMT, postmenopausal with hormone replacement therapy; SBP, systolic blood pressure; TG, triglycerides; WHO, World Health Organization; WHR, waist-to-hip ratio.

Sources:

1. Wang et al·, 2013^1^
2. Systematic search
3. This study

**eTable 3. Systematic literature review search terms.** The following terms were searched in the PubMed database to identify prospective studies that have investigated the association between IL-6 levels and incident type 2 diabetes.

|  | Search Terms |
| --- | --- |
| [#](http://www.ncbi.nlm.nih.gov/pubmed/advanced)5 | #1 AND #2 AND #3 AND #4 |
| [#4](http://www.ncbi.nlm.nih.gov/pubmed/advanced) | "2012/02/10"[Date - Publication] : "2018/10/30"[Date - Publication] |
| [#3](http://www.ncbi.nlm.nih.gov/pubmed/advanced) | "Cohort Studies"[MeSH] OR Cohort Study OR Studies, Cohort OR Study, Cohort OR Studies, Historical Cohort OR Cohort Studies, Historical Cohort Study, Historical OR Historical Cohort Study OR Study, Historical OR Analysis, Cohort OR Analyses, Cohort OR Cohort Analyses OR Cohort Analysis OR Incidence Studies OR Incidence Study OR Studies, Incidence OR Study, Incidence OR "Incidence"[MeSH] OR "Cohort Studies"[MeSH] OR Case-cohort study OR Nested case-control study OR Survey |
| [#2](http://www.ncbi.nlm.nih.gov/pubmed/advanced) | Diabetes Mellitus, Type 2[MeSH] OR NIDDM OR Maturity-Onset Diabetes OR Diabetes Mellitus, Noninsulin-Dependent OR Diabetes Mellitus, Adult-Onset OR Adult-Onset Diabetes Mellitus OR Diabetes Mellitus, Adult Onset OR Diabetes Mellitus, Ketosis-Resistant OR Diabetes Mellitus, Ketosis Resistant OR Ketosis-Resistant Diabetes Mellitus OR Diabetes Mellitus, Maturity-Onset OR Diabetes Mellitus, Maturity Onset OR Diabetes Mellitus, Non-Insulin Dependent OR Diabetes Mellitus, Non-Insulin-Dependent OR Non-Insulin-Dependent Diabetes Mellitus OR Diabetes Mellitus, Noninsulin Dependent OR Diabetes Mellitus, Slow-Onset OR Diabetes Mellitus, Slow Onset OR Slow-Onset Diabetes Mellitus OR Diabetes Mellitus, Stable OR Stable Diabetes Mellitus OR Diabetes Mellitus, Type II OR Maturity-Onset Diabetes Mellitus OR Maturity Onset Diabetes Mellitus OR MODY OR Type 2 Diabetes Mellitus OR Noninsulin-Dependent Diabetes Mellitus OR T2D OR T2DM OR Type 2 Diabetes[tiab] OR Type 2 diabetes mellitus OR diabetes[ti] |
| [#1](http://www.ncbi.nlm.nih.gov/pubmed/advanced) | Interleukin-6[MeSH] OR Interleukin 6 OR IL6 OR B-Cell Stimulatory Factor 2 OR B-Cell Stimulatory Factor-2 OR Differentiation Factor-2, B-Cell OR Differentiation Factor 2, B Cell OR B-Cell Differentiation Factor-2 OR B Cell Differentiation Factor 2 OR BSF-2 OR Hybridoma Growth Factor OR Growth Factor, Hybridoma OR IFN-beta 2 OR Plasmacytoma Growth Factor OR Growth Factor, Plasmacytoma OR Hepatocyte-Stimulating Factor OR Hepatocyte Stimulating Factor OR MGI-2 OR Myeloid Differentiation-Inducing Protein OR Differentiation-Inducing Protein, Myeloid OR Myeloid Differentiation Inducing Protein OR B-Cell Differentiation Factor OR B Cell Differentiation Factor OR Differentiation Factor, B-Cell OR Differentiation Factor, B Cell OR IL-6 OR Interferon beta-2 OR Interferon beta 2 OR beta-2, Interferon OR B Cell Stimulatory Factor-2 OR B Cell Stimulatory Factor 2 |

**eTable 4. Quality ratings of the studies included in the meta-analysis of association between IL-6 levels and incident type 2 diabetes.**

| **Study, year** | **Size and representativeness** | **Reliably measured exposure** | **Ascertainment of diabetes** | **Adjustment for possible confounders** | **Overall quality score** |
| --- | --- | --- | --- | --- | --- |
| Pradhan *et al.* 2001 | 1 | 1 | 1 | 2 | 5 |
| Duncan *et al.* 2003 | 1 | 1 | 1 | 1 | 4 |
| Krakoff *et al.* 2003 | 0 | 1 | 1 | 1 | 3 |
| Spranger *et al.* 2003 | 1 | 1 | 1 | 1 | 4 |
| Hu *et al.* 2004 | 1 | 1 | 0 | 2 | 4 |
| Liu *et al.* 2007 | 1 | 1 | 0 | 1 | 3 |
| Thorand *et al.* 2007 M ^a^ | 1 | 0 | 1 | 2 | 4 |
| Thorand *et al.* 2007 F ^a^ | 1 | 0 | 1 | 2 | 4 |
| Wannamethee *et al.* 2007 | 1 | 1 | 1 | 1 | 4 |
| Ley *et al.* 2008 | 0 | 1 | 1 | 1 | 3 |
| Bertoni *et al.* 2010 | 1 | 1 | 1 | 1 | 4 |
| Dallmeier *et al.* 2012 | 1 | 1 | 1 | 1 | 4 |
| Marques-Vidal *et al.* 2012 | 1 | 1 | 1 | 1 | 4 |
| Koloverou *et al.* 2018 | 1 | 1 | 1 | 2 | 5 |
| EPIC-Norfolk, 2018 | 1 | 1 | 2 | 2 | 6 |

Abbreviations: M, Males; F, Females; EPIC, European prospective investigation of cancer

1. IL-6 levels were measured differently between males aged 45-64 years compared to males aged 35-44 years and all females in the study therefore estimates were analysed separately.

Each study received up to 6 points based on the criteria below:

1. Size and representativeness: the study sample was representative of participants (i.e. sample size was more than 500, or more than 80% of eligible participants were invited, or more than 80% agreed to participate). [1 point]
2. Reliably measured exposure (ELISA = 1, Electrochemiluminescent assay = 1, particle-based flow cytometry = 1, light-scatter based methods other than flow cytometry = 0). [1 point]
3. The study reliably assessed outcome (Only self-reported = 0, objective glycaemic measures = +1 point to score, use of health records = +1 point to score). [2 points]
4. Adjustment for possible confounders (Adjusted only for age and sex = 0, Adjusted for age, sex and self-reported risk factors = 1, additionally adjusted for anthropometry = 2, adjusted for glycaemic measures = -1 point from score) [2 points]

The arbitrary categorization of overall quality score was low (0 to 2 points), good (3 or 4 points), or high (5 or 6 points).

**eTable 5. Fenland participant DEXA and glycemic measures.**

| **Variable** | **Value** |
| --- | --- |
| Country | United Kingdom |
| Number of participants | 10,344 |
| Baseline age, mean years (SD) | 48 (8) |
| Female sex, N (%) | 5,528 (53) |
| Arms fat mass, median (IQR) in grams | 2,516 (2,046-3,099) |
| Trunk fat mass, median (IQR) in grams | 13,142 (9,570-17,268) |
| Abdominal fat mass total, median (IQR) in grams | 2,145 (1,435-2,991) |
| Subcutaneous abdominal fat mass, median (IQR) in grams | 1,239 (921-1,667) |
| Visceral abdominal fat mass, median (IQR) in grams | 766 (317-1,409) |
| Gluteofemoral fat mass, median (IQR) in grams | 4,124 (3,298-5,174) |
| Leg fat mass, median (IQR) in grams | 8,285 (6,652-10,446) |
| Fasting glucose, mean (SD) in mmol/L | 4·83 (0·66) |
| 2-hour glucose, mean (SD) in mmol/L | 5·29 (1·70) |
| HbA1c, mean (SD) in % | 5·54 (0·46) |
| Fasting insulin, median (IQR) in pmol/L | 38·3 (26·5-57·2) |

Abbreviations: SD, Standard deviation; N, Number of participants; IQR, Interquartile range; mmol/L, millimoles per Litre; pmol/L, picomoles per Litre; HbA1c, glycated haemoglobin

**eTable 6. Standard deviation values used to convert estimates for continuous metabolic traits between clinical and standardized units and their source.**

| **Metabolic trait** | **Clinical units** | **Standard deviation** | **Source study** |
| --- | --- | --- | --- |
| 2-hour glucose | mmol/L | 1·7 | Fenland |
| Fasting plasma glucose | mmol/L | 0·66 | Fenland |
| HbA1c | % | 0·49 | Fenland |
| Fasting insulin | Log (pmol/L) | 0·60 | Fenland |
| Non-fasted glucose | mmol/L | 0·87 | UK Biobank |
| BMI | kg/m2 | 4·8 | UK Biobank |
| Hip circumference | cm | 9·2 | UK Biobank |
| Waist circumference | cm | 13·5 | UK Biobank |
| WHRadjBMI | ratio | 0·056 | UK Biobank |
| WHR | ratio | 0·09 | UK Biobank |

Abbreviations: BMI, Body mass index; WHR, Waist-to-hip ratio; WHRadjBMI, WHR adjusted for BMI; mmol, millimoles; L, Litre; pmol, picomoles; Kg, Kilograms; m, meters; cm, centimetres

Variables with a skewed distribution were normalized using the natural log transformation.

**eTable 7. Polygenic risk scores used in Stage 2 and their sources·**

| **Phenotype** | **SNP ID** | **EA ^a^** | **Beta** | **PMID** |
| --- | --- | --- | --- | --- |
| BMI | rs543874 | A | 0·205 | 25673413^14^ |
| BMI | rs3101336 | T | 0·395 |  |
| BMI | rs12566985 | G | 0·450 |  |
| BMI | rs17024393 | T | 0·026 |  |
| BMI | rs657452 | A | 0·399 |  |
| BMI | rs11165643 | C | 0·419 |  |
| BMI | rs12401738 | G | 0·366 |  |
| BMI | rs2820292 | A | 0·441 |  |
| BMI | rs11583200 | C | 0·399 |  |
| BMI | rs977747 | T | 0·426 |  |
| BMI | rs7903146 | C | 0·290 |  |
| BMI | rs17094222 | T | 0·209 |  |
| BMI | rs11191560 | T | 0·081 |  |
| BMI | rs7899106 | A | 0·051 |  |
| BMI | rs11030104 | A | 0·203 |  |
| BMI | rs3817334 | C | 0·402 |  |
| BMI | rs12286929 | A | 0·476 |  |
| BMI | rs4256980 | C | 0·354 |  |
| BMI | rs2176598 | T | 0·248 |  |
| BMI | rs7138803 | G | 0·365 |  |
| BMI | rs11057405 | G | 0·100 |  |
| BMI | rs12429545 | G | 0·130 |  |
| BMI | rs9581854 | C | 0·175 |  |
| BMI | rs1441264 | G | 0·402 |  |
| BMI | rs9540493 | A | 0·461 |  |
| BMI | rs7141420 | C | 0·481 |  |
| BMI | rs10132280 | C | 0·303 |  |
| BMI | rs12885454 | C | 0·352 |  |
| BMI | rs11847697 | C | 0·051 |  |
| BMI | rs16951275 | T | 0·235 |  |
| BMI | rs7164727 | C | 0·343 |  |
| BMI | rs3736485 | A | 0·468 |  |
| BMI | rs1558902 | T | 0·394 |  |
| BMI | rs3888190 | C | 0·390 |  |
| BMI | rs12446632 | G | 0·138 |  |
| BMI | rs758747 | C | 0·287 |  |
| BMI | rs9925964 | A | 0·354 |  |
| BMI | rs2650492 | G | 0·288 |  |
| BMI | rs2080454 | C | 0·390 |  |
| BMI | rs4787491 | A | 0·465 |  |
| BMI | rs12940622 | G | 0·439 |  |
| BMI | rs1000940 | A | 0·305 |  |
| BMI | rs9914578 | C | 0·213 |  |
| BMI | rs6567160 | T | 0·235 |  |
| BMI | rs7239883 | G | 0·377 |  |
| BMI | rs7243357 | T | 0·178 |  |
| BMI | rs1808579 | C | 0·482 |  |
| BMI | rs2287019 | C | 0·181 |  |
| BMI | rs3810291 | G | 0·342 |  |
| BMI | rs2075650 | A | 0·144 |  |
| BMI | rs29941 | A | 0·326 |  |
| BMI | rs17724992 | A | 0·270 |  |
| BMI | rs13021737 | A | 0·169 |  |
| BMI | rs10182181 | A | 0·493 |  |
| BMI | rs1016287 | T | 0·295 |  |
| BMI | rs7599312 | G | 0·266 |  |
| BMI | rs11126666 | G | 0·258 |  |
| BMI | rs492400 | C | 0·429 |  |
| BMI | rs2176040 | A | 0·348 |  |
| BMI | rs1528435 | C | 0·378 |  |
| BMI | rs11688816 | G | 0·457 |  |
| BMI | rs2121279 | C | 0·121 |  |
| BMI | rs17203016 | A | 0·196 |  |
| BMI | rs1460676 | T | 0·159 |  |
| BMI | rs6091540 | C | 0·295 |  |
| BMI | rs2836754 | T | 0·377 |  |
| BMI | rs1516725 | T | 0·138 |  |
| BMI | rs13078960 | T | 0·195 |  |
| BMI | rs2365389 | C | 0·422 |  |
| BMI | rs16851483 | G | 0·067 |  |
| BMI | rs6804842 | A | 0·432 |  |
| BMI | rs3849570 | C | 0·350 |  |
| BMI | rs10938397 | A | 0·429 |  |
| BMI | rs13107325 | C | 0·072 |  |
| BMI | rs17001654 | C | 0·148 |  |
| BMI | rs11727676 | T | 0·093 |  |
| BMI | rs2112347 | T | 0·368 |  |
| BMI | rs7715256 | G | 0·430 |  |
| BMI | rs2207139 | A | 0·169 |  |
| BMI | rs205262 | A | 0·275 |  |
| BMI | rs13191362 | A | 0·122 |  |
| BMI | rs751414 | G | 0·283 |  |
| BMI | rs9400239 | T | 0·309 |  |
| BMI | rs9374842 | C | 0·231 |  |
| BMI | rs13201877 | A | 0·130 |  |
| BMI | rs9641123 | G | 0·399 |  |
| BMI | rs1167827 | A | 0·431 |  |
| BMI | rs2245368 | C | 0·176 |  |
| BMI | rs6465468 | G | 0·299 |  |
| BMI | rs17405819 | T | 0·295 |  |
| BMI | rs16907751 | C | 0·099 |  |
| BMI | rs2033732 | T | 0·252 |  |
| BMI | rs10968576 | A | 0·315 |  |
| BMI | rs1928295 | T | 0·430 |  |
| BMI | rs4740619 | T | 0·453 |  |
| BMI | rs10733682 | A | 0·473 |  |
| BMI | rs6477694 | C | 0·359 |  |
| WHRadjBMI | rs998584 | A | 0·05 | 30575882^6^ |
| WHRadjBMI | rs1936805 | T | 0·041 |  |
| WHRadjBMI | rs7133378 | G | 0·039 |  |
| WHRadjBMI | rs2371767 | G | 0·04 |  |
| WHRadjBMI | rs2791550 | G | 0·037 |  |
| WHRadjBMI | rs10923724 | T | 0·035 |  |
| WHRadjBMI | rs10195252 | T | 0·031 |  |
| WHRadjBMI | rs1294410 | C | 0·031 |  |
| WHRadjBMI | rs718314 | G | 0·034 |  |
| WHRadjBMI | rs3786897 | G | 0·03 |  |
| WHRadjBMI | rs10919388 | C | 0·033 |  |
| WHRadjBMI | rs714515 | G | 0·028 |  |
| WHRadjBMI | rs17451107 | T | 0·029 |  |
| WHRadjBMI | rs2236519 | A | 0·03 |  |
| WHRadjBMI | rs6861681 | A | 0·029 |  |
| WHRadjBMI | rs1443512 | A | 0·031 |  |
| WHRadjBMI | rs9837325 | C | 0·034 |  |
| WHRadjBMI | rs459193 | A | 0·029 |  |
| WHRadjBMI | rs2167750 | T | 0·027 |  |
| WHRadjBMI | rs12608504 | A | 0·026 |  |
| WHRadjBMI | rs2145272 | G | 0·026 |  |
| WHRadjBMI | rs2294239 | A | 0·025 |  |
| WHRadjBMI | rs797486 | A | 0·037 |  |
| WHRadjBMI | rs1055144 | T | 0·03 |  |
| WHRadjBMI | rs605203 | A | 0·025 |  |
| WHRadjBMI | rs12214804 | C | 0·045 |  |
| WHRadjBMI | rs4738141 | G | 0·028 |  |
| WHRadjBMI | rs2276824 | C | 0·024 |  |
| WHRadjBMI | rs17819328 | G | 0·023 |  |
| WHRadjBMI | rs634869 | T | 0·023 |  |
| WHRadjBMI | rs2925979 | T | 0·024 |  |
| WHRadjBMI | rs905938 | T | 0·025 |  |
| WHRadjBMI | rs8066985 | A | 0·022 |  |
| WHRadjBMI | rs10502148 | C | 0·023 |  |
| WHRadjBMI | rs1569135 | A | 0·021 |  |
| WHRadjBMI | rs7492628 | G | 0·024 |  |
| WHRadjBMI | rs6556301 | T | 0·022 |  |
| WHRadjBMI | rs9792666 | A | 0·057 |  |
| WHRadjBMI | rs2845885 | C | 0·045 |  |
| WHRadjBMI | rs3810068 | T | 0·023 |  |
| WHRadjBMI | rs7801581 | T | 0·023 |  |
| WHRadjBMI | rs12936587 | G | 0·02 |  |
| WHRadjBMI | rs3747577 | C | 0·023 |  |
| WHRadjBMI | rs757608 | A | 0·02 |  |
| WHRadjBMI | rs143384 | A | 0·02 |  |
| WHRadjBMI | rs951252 | G | 0·019 |  |
| WHRadjBMI | rs2428549 | G | 0·021 |  |
| WHRadjBMI | rs2073267 | G | 0·025 |  |
| WHRadjBMI | rs601339 | A | 0·025 |  |
| WHRadjBMI | rs910382 | G | 0·019 |  |
| WHRadjBMI | rs711869 | G | 0·019 |  |
| WHRadjBMI | rs1051921 | G | 0·023 |  |
| WHRadjBMI | rs11263432 | T | 0·02 |  |
| WHRadjBMI | rs7598832 | C | 0·019 |  |
| WHRadjBMI | rs6719672 | G | 0·025 |  |
| WHRadjBMI | rs780159 | G | 0·018 |  |
| WHRadjBMI | rs6688233 | T | 0·022 |  |
| WHRadjBMI | rs622217 | T | 0·019 |  |
| WHRadjBMI | rs16891532 | A | 0·034 |  |
| WHRadjBMI | rs9644033 | A | 0·022 |  |
| WHRadjBMI | rs10264590 | A | 0·018 |  |
| WHRadjBMI | rs6446204 | C | 0·02 |  |
| WHRadjBMI | rs10462028 | A | 0·019 |  |
| WHRadjBMI | rs8030605 | A | 0·026 |  |
| WHRadjBMI | rs10992408 | G | 0·024 |  |
| WHRadjBMI | rs4779526 | A | 0·021 |  |
| WHRadjBMI | rs4727695 | A | 0·029 |  |
| WHRadjBMI | rs6853254 | T | 0·019 |  |
| WHRadjBMI | rs9388766 | C | 0·018 |  |
| WHRadjBMI | rs6581662 | T | 0·02 |  |
| WHRadjBMI | rs3741378 | C | 0·024 |  |
| WHRadjBMI | rs13256367 | A | 0·018 |  |
| WHRadjBMI | rs2993481 | T | 0·021 |  |
| WHRadjBMI | rs11724804 | G | 0·017 |  |
| WHRadjBMI | rs4420638 | A | 0·022 |  |
| WHRadjBMI | rs1045241 | C | 0·018 |  |
| WHRadjBMI | rs4450871 | A | 0·018 |  |
| WHRadjBMI | rs7589318 | G | 0·017 |  |
| WHRadjBMI | rs2836179 | G | 0·017 |  |
| WHRadjBMI | rs11592754 | C | 0·023 |  |
| WHRadjBMI | rs303084 | A | 0·019 |  |
| WHRadjBMI | rs9844972 | C | 0·033 |  |
| WHRadjBMI | rs2047937 | C | 0·016 |  |
| WHRadjBMI | rs3764002 | C | 0·018 |  |
| WHRadjBMI | rs727428 | T | 0·016 |  |
| WHRadjBMI | rs10980802 | G | 0·016 |  |
| WHRadjBMI | rs11747001 | A | 0·018 |  |
| WHRadjBMI | rs2222543 | G | 0·016 |  |
| WHRadjBMI | rs39312 | C | 0·016 |  |
| WHRadjBMI | rs7235010 | A | 0·018 |  |
| WHRadjBMI | rs9362097 | G | 0·016 |  |
| WHRadjBMI | rs9583489 | C | 0·017 |  |
| WHRadjBMI | rs1805741 | C | 0·017 |  |
| WHRadjBMI | rs2254069 | A | 0·024 |  |
| WHRadjBMI | rs2235529 | C | 0·021 |  |
| WHRadjBMI | rs17041868 | C | 0·03 |  |
| WHRadjBMI | rs13154197 | G | 0·027 |  |
| WHRadjBMI | rs2298632 | C | 0·015 |  |
| WHRadjBMI | rs1876829 | C | 0·019 |  |
| WHRadjBMI | rs1440372 | C | 0·016 |  |
| WHRadjBMI | rs4902632 | A | 0·021 |  |
| WHRadjBMI | rs6433219 | A | 0·017 |  |
| WHRadjBMI | rs7122422 | C | 0·015 |  |
| WHRadjBMI | rs2444770 | T | 0·021 |  |
| WHRadjBMI | rs10512606 | C | 0·027 |  |
| WHRadjBMI | rs11079041 | A | 0·016 |  |
| WHRadjBMI | rs4849294 | T | 0·015 |  |
| WHRadjBMI | rs1316979 | T | 0·029 |  |
| WHRadjBMI | rs4851221 | G | 0·019 |  |
| WHRadjBMI | rs1144 | C | 0·015 |  |
| WHRadjBMI | rs3851294 | G | 0·025 |  |
| WHRadjBMI | rs1105881 | G | 0·015 |  |
| WHRadjBMI | rs11051005 | A | 0·016 |  |
| WHRadjBMI | rs6920788 | T | 0·016 |  |
| WHRadjBMI | rs7680787 | T | 0·014 |  |
| WHRadjBMI | rs12459350 | A | 0·014 |  |
| WHRadjBMI | rs4704389 | A | 0·014 |  |
| WHRadjBMI | rs6932767 | T | 0·017 |  |
| WHRadjBMI | rs1474921 | A | 0·015 |  |
| WHRadjBMI | rs380654 | G | 0·014 |  |
| WHRadjBMI | rs8055190 | C | 0·034 |  |
| WHRadjBMI | rs9647379 | G | 0·014 |  |
| WHRadjBMI | rs2398893 | A | 0·015 |  |
| WHRadjBMI | rs2821391 | A | 0·015 |  |
| WHRadjBMI | rs998749 | A | 0·013 |  |
| WHRadjBMI | rs12828318 | A | 0·018 |  |
| WHRadjBMI | rs10844642 | A | 0·014 |  |
| WHRadjBMI | rs1328757 | T | 0·014 |  |
| WHRadjBMI | rs1053593 | G | 0·014 |  |
| WHRadjBMI | rs1498126 | C | 0·017 |  |
| WHRadjBMI | rs1190982 | T | 0·015 |  |
| WHRadjBMI | rs12774134 | C | 0·02 |  |
| WHRadjBMI | rs11726981 | C | 0·015 |  |
| WHRadjBMI | rs7800072 | G | 0·014 |  |
| WHRadjBMI | rs10880321 | G | 0·014 |  |
| WHRadjBMI | rs7823561 | A | 0·014 |  |
| WHRadjBMI | rs4751628 | G | 0·014 |  |
| WHRadjBMI | rs7242873 | G | 0·024 |  |
| WHRadjBMI | rs2061705 | G | 0·013 |  |
| WHRadjBMI | rs17326656 | T | 0·015 |  |
| WHRadjBMI | rs13406302 | C | 0·014 |  |
| WHRadjBMI | rs2452877 | A | 0·013 |  |
| WHRadjBMI | rs7114403 | A | 0·013 |  |
| WHRadjBMI | rs2283847 | T | 0·013 |  |
| WHRadjBMI | rs11187537 | C | 0·015 |  |
| WHRadjBMI | rs6752964 | C | 0·02 |  |
| WHRadjBMI | rs3789615 | C | 0·012 |  |
| WHRadjBMI | rs11893688 | T | 0·014 |  |
| WHRadjBMI | rs332105 | G | 0·013 |  |
| WHRadjBMI | rs8030277 | T | 0·013 |  |
| WHRadjBMI | rs10963067 | C | 0·022 |  |
| WHRadjBMI | rs174829 | G | 0·014 |  |
| WHRadjBMI | rs2057869 | A | 0·014 |  |
| WHRadjBMI | rs421168 | G | 0·013 |  |
| WHRadjBMI | rs10745659 | G | 0·012 |  |
| WHRadjBMI | rs7612999 | A | 0·014 |  |
| WHRadjBMI | rs6449133 | T | 0·013 |  |
| WHRadjBMI | rs2595004 | C | 0·016 |  |
| WHRadjBMI | rs7919055 | C | 0·032 |  |
| WHRadjBMI | rs2066107 | T | 0·013 |  |
| WHRadjBMI | rs2058914 | G | 0·013 |  |
| WHRadjBMI | rs2333496 | T | 0·012 |  |
| WHRadjBMI | rs10887759 | A | 0·016 |  |
| WHRadjBMI | rs12684047 | T | 0·016 |  |
| WHRadjBMI | rs4239275 | T | 0·012 |  |
| WHRadjBMI | rs6486060 | G | 0·012 |  |
| WHRadjBMI | rs2320125 | T | 0·012 |  |
| WHRadjBMI | rs807067 | T | 0·011 |  |
| WHRadjBMI | rs17167945 | G | 0·015 |  |
| WHRadjBMI | rs36232 | G | 0·015 |  |
| WHRadjBMI | rs9750952 | C | 0·014 |  |
| WHRadjBMI | rs710122 | G | 0·012 |  |
| WHRadjBMI | rs9896963 | C | 0·014 |  |
| WHRadjBMI | rs9305545 | G | 0·016 |  |
| WHRadjBMI | rs747249 | A | 0·012 |  |
| WHRadjBMI | rs6874524 | T | 0·013 |  |
| WHRadjBMI | rs676556 | G | 0·014 |  |
| WHRadjBMI | rs6908042 | A | 0·011 |  |
| WHRadjBMI | rs2701523 | A | 0·012 |  |
| WHRadjBMI | rs1360485 | T | 0·011 |  |
| WHRadjBMI | rs544668 | T | 0·011 |  |
| WHRadjBMI | rs7091853 | C | 0·011 |  |
| WHRadjBMI | rs1979527 | A | 0·013 |  |
| WHRadjBMI | rs1278493 | G | 0·011 |  |
| WHRadjBMI | rs12631066 | C | 0·013 |  |
| WHRadjBMI | rs2823096 | A | 0·014 |  |
| WHRadjBMI | rs10891483 | T | 0·017 |  |
| WHRadjBMI | rs362275 | C | 0·011 |  |
| WHRadjBMI | rs6496127 | G | 0·01 |  |
| WHRadjBMI | rs7235891 | C | 0·011 |  |
| WHRadjBMI | rs1156979 | A | 0·011 |  |
| WHRadjBMI | rs505102 | C | 0·013 |  |
| WHRadjBMI | rs10507524 | C | 0·018 |  |
| WHRadjBMI | rs13028903 | T | 0·011 |  |
| WHRadjBMI | rs12440695 | C | 0·01 |  |
| WHRadjBMI | rs2590440 | G | 0·013 |  |
| WHRadjBMI | rs2240328 | T | 0·014 |  |
| WHRadjBMI | rs3758938 | T | 0·011 |  |
| WHRadjBMI | rs4454603 | C | 0·01 |  |
| WHRadjBMI | rs1328369 | T | 0·01 |  |
| WHRadjBMI | rs12186798 | G | 0·013 |  |
| WHRadjBMI | rs15285 | C | 0·011 |  |
| Hip-specific score for higher WHR | rs10195252 | T | 0·031 |  |
| Hip-specific score for higher WHR | rs9837325 | C | 0·034 |  |
| Hip-specific score for higher WHR | rs2167750 | T | 0·027 |  |
| Hip-specific score for higher WHR | rs2145272 | G | 0·026 |  |
| Hip-specific score for higher WHR | rs605203 | A | 0·025 |  |
| Hip-specific score for higher WHR | rs2845885 | C | 0·045 |  |
| Hip-specific score for higher WHR | rs12936587 | G | 0·02 |  |
| Hip-specific score for higher WHR | rs3747577 | C | 0·023 |  |
| Hip-specific score for higher WHR | rs601339 | A | 0·025 |  |
| Hip-specific score for higher WHR | rs10992408 | G | 0·024 |  |
| Hip-specific score for higher WHR | rs4450871 | A | 0·018 |  |
| Hip-specific score for higher WHR | rs11592754 | C | 0·023 |  |
| Hip-specific score for higher WHR | rs727428 | T | 0·016 |  |
| Hip-specific score for higher WHR | rs2235529 | C | 0·021 |  |
| Hip-specific score for higher WHR | rs13154197 | G | 0·027 |  |
| Hip-specific score for higher WHR | rs7122422 | C | 0·015 |  |
| Hip-specific score for higher WHR | rs1316979 | T | 0·029 |  |
| Hip-specific score for higher WHR | rs6920788 | T | 0·016 |  |
| Hip-specific score for higher WHR | rs12459350 | A | 0·014 |  |
| Hip-specific score for higher WHR | rs4704389 | A | 0·014 |  |
| Hip-specific score for higher WHR | rs2061705 | G | 0·013 |  |
| Hip-specific score for higher WHR | rs2320125 | T | 0·012 |  |
| Waist-specific score for higher WHR | rs17451107 | T | 0·029 |  |
| Waist-specific score for higher WHR | rs1055144 | T | 0·03 |  |
| Waist-specific score for higher WHR | rs1569135 | A | 0·021 |  |
| Waist-specific score for higher WHR | rs757608 | A | 0·02 |  |
| Waist-specific score for higher WHR | rs7598832 | C | 0·019 |  |
| Waist-specific score for higher WHR | rs6719672 | G | 0·025 |  |
| Waist-specific score for higher WHR | rs11724804 | G | 0·017 |  |
| Waist-specific score for higher WHR | rs11747001 | A | 0·018 |  |
| Waist-specific score for higher WHR | rs39312 | C | 0·016 |  |
| Waist-specific score for higher WHR | rs9362097 | G | 0·016 |  |
| Waist-specific score for higher WHR | rs11079041 | A | 0·016 |  |
| Waist-specific score for higher WHR | rs4849294 | T | 0·015 |  |
| Waist-specific score for higher WHR | rs6932767 | T | 0·017 |  |
| Waist-specific score for higher WHR | rs9647379 | G | 0·014 |  |
| Waist-specific score for higher WHR | rs998749 | A | 0·013 |  |
| Waist-specific score for higher WHR | rs10844642 | A | 0·014 |  |
| Waist-specific score for higher WHR | rs17326656 | T | 0·015 |  |
| Waist-specific score for higher WHR | rs6752964 | C | 0·02 |  |
| Waist-specific score for higher WHR | rs11893688 | T | 0·014 |  |
| Waist-specific score for higher WHR | rs332105 | G | 0·013 |  |
| Waist-specific score for higher WHR | rs10963067 | C | 0·022 |  |
| Waist-specific score for higher WHR | rs2058914 | G | 0·013 |  |
| Waist-specific score for higher WHR | rs710122 | G | 0·012 |  |
| Waist-specific score for higher WHR | rs9305545 | G | 0·016 |  |
| Waist-specific score for higher WHR | rs676556 | G | 0·014 |  |
| Waist-specific score for higher WHR | rs6908042 | A | 0·011 |  |
| Waist-specific score for higher WHR | rs544668 | T | 0·011 |  |
| Waist-specific score for higher WHR | rs12631066 | C | 0·013 |  |
| Waist-specific score for higher WHR | rs2823096 | A | 0·014 |  |
| Waist-specific score for higher WHR | rs10891483 | T | 0·017 |  |
| Waist-specific score for higher WHR | rs362275 | C | 0·011 |  |
| Waist-specific score for higher WHR | rs13028903 | T | 0·011 |  |
| Waist-specific score for higher WHR | rs2590440 | G | 0·013 |  |
| Waist-specific score for higher WHR | rs2240328 | T | 0·014 |  |
| Waist-specific score for higher WHR | rs4454603 | C | 0·01 |  |
| Waist-specific score for higher WHR | rs1328369 | T | 0·01 |  |
| HbA1c via glycemic mechanisms | rs560887 | C | 0·0284 | 28898252^15^ |
| HbA1c via glycemic mechanisms | rs11708067 | A | 0·0132 |  |
| HbA1c via glycemic mechanisms | rs8192675 | T | 0·0112 |  |
| HbA1c via glycemic mechanisms | rs13134327 | A | 0·0131 |  |
| HbA1c via glycemic mechanisms | rs7756992 | G | 0·0123 |  |
| HbA1c via glycemic mechanisms | rs4607517 | A | 0·0306 |  |
| HbA1c via glycemic mechanisms | rs11558471 | A | 0·015 |  |
| HbA1c via glycemic mechanisms | rs2383208 | A | 0·0142 |  |
| HbA1c via glycemic mechanisms | rs579459 | C | 0·0107 |  |
| HbA1c via glycemic mechanisms | rs17747324 | C | 0·0149 |  |
| HbA1c via glycemic mechanisms | rs11603334 | G | 0·012 |  |
| HbA1c via glycemic mechanisms | rs10830963 | G | 0·0196 |  |
| Fasting plasma glucose | rs10438234 | C | 0·013 | 22581228^16^; 22885924^17^; 24947364^18^ |
| Fasting plasma glucose | rs10747083 | A | 0·013 |  |
| Fasting plasma glucose | rs10811661 | T | 0·024 |  |
| Fasting plasma glucose | rs10814916 | C | 0·016 |  |
| Fasting plasma glucose | rs10830963 | G | 0·078 |  |
| Fasting plasma glucose | rs11039149 | A | 0·025 |  |
| Fasting plasma glucose | rs11558471 | A | 0·029 |  |
| Fasting plasma glucose | rs11603334 | G | 0·019 |  |
| Fasting plasma glucose | rs11607883 | G | 0·021 |  |
| Fasting plasma glucose | rs11619319 | G | 0·02 |  |
| Fasting plasma glucose | rs11708067 | A | 0·023 |  |
| Fasting plasma glucose | rs1280 | T | 0·026 |  |
| Fasting plasma glucose | rs1334577 | A | 0·013 |  |
| Fasting plasma glucose | rs16913693 | T | 0·043 |  |
| Fasting plasma glucose | rs17264369 | A | 0·017 |  |
| Fasting plasma glucose | rs174576 | C | 0·02 |  |
| Fasting plasma glucose | rs2191348 | T | 0·029 |  |
| Fasting plasma glucose | rs2302593 | C | 0·014 |  |
| Fasting plasma glucose | rs2657879 | G | 0·012 |  |
| Fasting plasma glucose | rs340874 | C | 0·013 |  |
| Fasting plasma glucose | rs3829109 | G | 0·017 |  |
| Fasting plasma glucose | rs4258313 | G | 0·035 |  |
| Fasting plasma glucose | rs4502156 | T | 0·022 |  |
| Fasting plasma glucose | rs4869272 | T | 0·018 |  |
| Fasting plasma glucose | rs573225 | A | 0·063 |  |
| Fasting plasma glucose | rs576674 | G | 0·017 |  |
| Fasting plasma glucose | rs6113722 | G | 0·035 |  |
| Fasting plasma glucose | rs6943153 | T | 0·015 |  |
| Fasting plasma glucose | rs730497 | A | 0·057 |  |
| Fasting plasma glucose | rs7651090 | G | 0·013 |  |
| Fasting plasma glucose | rs7708285 | G | 0·011 |  |
| Fasting plasma glucose | rs7756992 | G | 0·014 |  |
| Fasting plasma glucose | rs780093 | C | 0·028 |  |
| Fasting plasma glucose | rs7903146 | T | 0·022 |  |
| Fasting plasma glucose | rs9814873 | A | 0·0098 |  |
| Fasting plasma glucose | rs983309 | T | 0·026 |  |
| Fasting plasma glucose | rs10305492 | G | 0·071 |  |
| Fasting plasma glucose | rs651007 | A | 0·0099 |  |
| Impaired insulin secretion | rs10811661 | T | 0·083 |  |
| Impaired insulin secretion | rs10830963 | G | 0·076 |  |
| Impaired insulin secretion | rs10946398 | C | 0·061 |  |
| Impaired insulin secretion | rs11603334 | G | 0·051 |  |
| Impaired insulin secretion | rs11605924 | A | 0·069 |  |
| Impaired insulin secretion | rs11672660 | T | 0·13 |  |
| Impaired insulin secretion | rs12686676 | G | 0·015 |  |
| Impaired insulin secretion | rs12779790 | G | 0·068 |  |
| Impaired insulin secretion | rs13266634 | C | 0·041 |  |
| Impaired insulin secretion | rs174550 | T | 0·031 |  |
| Impaired insulin secretion | rs4502156 | T | 0·05 |  |
| Impaired insulin secretion | rs4607517 | A | 0·032 |  |
| Impaired insulin secretion | rs5015480 | C | 0·061 |  |
| Impaired insulin secretion | rs5219 | T | 0·038 |  |
| Impaired insulin secretion | rs560887 | T | 0·047 |  |
| Impaired insulin secretion | rs7903146 | T | 0·06 |  |
| Impaired insulin secretion | rs7957197 | T | 0·062 |  |
| Impaired insulin secretion | rs933360 | T | 0·079 |  |
| Insulin resistance Scott | rs10195252 | T | 0·017 |  |
| Insulin resistance Scott | rs17036328 | T | 0·021 |  |
| Insulin resistance Scott | rs2745353 | T | 0·011 |  |
| Insulin resistance Scott | rs2943645 | T | 0·019 |  |
| Insulin resistance Scott | rs3822072 | A | 0·012 |  |
| Insulin resistance Scott | rs459193 | G | 0·015 |  |
| Insulin resistance Scott | rs4846565 | G | 0·013 |  |
| Insulin resistance Scott | rs4865796 | A | 0·015 |  |
| Insulin resistance Scott | rs6536208 | A | 0·017 |  |
| Insulin resistance Scott | rs731839 | G | 0·015 |  |
| Insulin resistance | rs9425291 | A | 1 |  |
| Insulin resistance | rs7973683 | C | 1 |  |
| Insulin resistance | rs2745353 | T | 1 |  |
| Insulin resistance | rs2943645 | T | 1 |  |
| Insulin resistance | rs1045241 | C | 1 |  |
| Insulin resistance | rs8032586 | C | 1 |  |
| Insulin resistance | rs6937438 | A | 1 |  |
| Insulin resistance | rs4865796 | A | 1 |  |
| Insulin resistance | rs11231693 | A | 1 |  |
| Insulin resistance | rs10195252 | T | 1 |  |
| Insulin resistance | rs4738141 | G | 1 |  |
| Insulin resistance | rs3822072 | A | 1 |  |
| Insulin resistance | rs492400 | T | 1 |  |
| Insulin resistance | rs6066149 | G | 1 |  |
| Insulin resistance | rs966544 | G | 1 |  |
| Insulin resistance | rs17386142 | C | 1 |  |
| Insulin resistance | rs2126259 | T | 1 |  |
| Insulin resistance | rs11577194 | T | 1 |  |
| Insulin resistance | rs11130329 | A | 1 |  |
| Insulin resistance | rs6536208 | A | 1 |  |
| Insulin resistance | rs308971 | G | 1 |  |
| Insulin resistance | rs12525532 | T | 1 |  |
| Insulin resistance | rs731839 | G | 1 |  |
| Insulin resistance | rs459193 | G | 1 |  |
| Insulin resistance | rs718314 | G | 1 |  |
| Insulin resistance | rs4846565 | G | 1 |  |
| Insulin resistance | rs2249105 | A | 1 |  |
| Insulin resistance | rs2434612 | G | 1 |  |
| Insulin resistance | rs6887914 | C | 1 |  |
| Insulin resistance | rs10995441 | G | 1 |  |
| Insulin resistance | rs4804311 | A | 1 |  |
| Insulin resistance | rs4976033 | G | 1 |  |
| Insulin resistance | rs8101064 | T | 1 |  |
| Insulin resistance | rs3861397 | G | 1 |  |
| Insulin resistance | rs972283 | G | 1 |  |
| Insulin resistance | rs7323406 | A | 1 |  |
| Insulin resistance | rs1011685 | C | 1 |  |
| Insulin resistance | rs132985 | C | 1 |  |
| Insulin resistance | rs7227237 | C | 1 |  |
| Insulin resistance | rs17169104 | G | 1 |  |
| Insulin resistance | rs2699429 | C | 1 |  |
| Insulin resistance | rs7176058 | A | 1 |  |
| Insulin resistance | rs645040 | T | 1 |  |
| Insulin resistance | rs9492443 | C | 1 |  |
| Insulin resistance | rs683135 | A | 1 |  |
| Insulin resistance | rs754814 | T | 1 |  |
| Insulin resistance | rs17402950 | G | 1 |  |
| Insulin resistance | rs7005992 | C | 1 |  |
| Insulin resistance | rs4804833 | A | 1 |  |
| Insulin resistance | rs498313 | A | 1 |  |
| Insulin resistance | rs295449 | A | 1 |  |
| Insulin resistance | rs3864041 | T | 1 |  |
| Insulin resistance | rs9881942 | A | 1 |  |
| *MC4R* β-arrestin biased GoF score | rs2229616 | T | 1 | 31002796^19^ |
| *MC4R* β -arrestin biased GoF score | rs52820871 | G | 1 |  |
| *MC4R* β -arrestin biased GoF score | affx89015531 | A | 1 |  |
| *MC4R* β -arrestin biased GoF score | affx89019383 | C | 1 |  |

Abbreviations: WHRadjBMI; Waist-to-hip ratio adjusted for BMI; BMI, Body mass index; HbA1c, glycated haemoglobin; *MC4R,* Melanocortin 4 Receptor; GoF, Gain-of-function, EA; Effect allele; PMID, PubMed identification number.

1. EA represents the trait-raising allele

**eTable 8. Correlation of IL-6 levels with DEXA adiposity measures in Fenland.**

| **Adiposity trait (units)^a^** | **Correlation with IL-6 levels ^b c^** | **P-value** |
| --- | --- | --- |
| **Waist-to-hip ratio** | 0·17 | <0·0001 |
| **BMI in kg/m^2^** | 0·25 | <0·0001 |
| **Body fat percentage in %** | 0·23 | <0·0001 |
| **Abdominal fat mass in grams** | 0·27 | <0·0001 |
| **Gluteofemoral fat mass in grams** | 0·20 | <0·0001 |
| **Leg fat mass in grams** | 0·18 | <0·0001 |
| **Abdominal to gluteofemoral fat mass ratio** | 0·17 | <0·0001 |
| **Peripheral fat mass in grams** | 0·20 | <0·0001 |
| **Visceral fat mass in grams** | 0·24 | <0·0001 |
| **Subcutaneous fat mass in grams** | 0·20 | <0·0001 |

Abbreviations: BMI, Body Mass Index; IL-6, interleukin-6.

Adiposity measures accounted for 9% of the variance in IL-6 levels

1. DEXA adiposity traits were inverse-rank normal transformed
2. IL-6 levels were natural log transformed (pg/mL)
3. Pearson’s correlation coefficients are reported

**eTable 9. Variance and heritability in type 2 diabetes and coronary disease occurrence explained by *IL6R* Asp358Ala.**

| **Outcome** | **Variance explained (%)** | **Heritability explained (%)** |
| --- | --- | --- |
| Type 2 diabetes | 0·007 | 0·04 |
| Coronary artery disease | 0·007 | 0·07 |

**eTable 10. Association of 358Ala with type 2 diabetes conditioning on rs2481065.**

| **Analysis** | **Type 2 diabetes cases** | **Controls** | **OR (95% CI)** | **P-value** |
| --- | --- | --- | --- | --- |
| Main | 22,182 | 424,361 | 0·96 (0·95-0·98) | 3x10^-4^ |
| Conditioned on rs2481065 | 22,182 | 424,361 | 0·97 (0·95-0·99) | 0·007 |

Abbreviations: OR, Odds ratio; CI, Confidence interval

**eFigure 1. Design of the observational and genetic mediation analyses.**

The cohorts in which each coefficient was estimated are reported in the inner part of each panel. **Panel A** illustrates the design of the observational mediation analysis, estimated in the EPIC-Norfolk cohort. **Panels B and C** illustrate the design of the genetic mediation analyses, for cardiometabolic disease and fasting insulin levels respectively estimated in the EPIC-Norfolk, Fenland UK Biobank and MAGIC cohorts.

**eFigure 2. Conditional quantile regression of CRP levels in EPIC-Norfolk.** The association of 358Ala with ln-CRP levels was estimated in EPIC-Norfolk. The ordinary least squares (OLS) regression line is shown in red with the corresponding 95% confidence interval denoted by the dashed red lines. The OLS beta estimate and p-value are provided within the plot. The beta estimates from conditional quantile regression per ln-CRP quantile are shown by the black line and the corresponding 95% confidence interval is shown by the grey ribbon. The blue line shows the meta-regression line. The meta-regression estimate, and P-value are provided within the plot. No statistically significant difference in the effect of Asp358Ala across CRP quantiles is shown by the meta-regression model.

Abbreviations: OLS, Ordinary least squares; MR, Meta-regression; CRP, C-reactive protein; mg, milligrams; L, Litre; Asp, Aspartic acid; Ala, Alanine.

**eFigure 3. Association of IL-6 levels in deciles with incident type 2 diabetes in EPIC-Norfolk.** Estimates were adjusted for age, sex, BMI, WHR, ethnicity, education level, family history of type 2 diabetes, smoking status, average units of alcohol per week and average physical activity per week. Hazard ratios for type 2 diabetes were estimated relative to the lowest decile.

Abbreviations: HR, Hazard ratio; CI, Confidence interval; BMI, Body mass index; WHR, Waist-to-hip ratio; T2D, Type 2 diabetes; Avg., Average; pg/mL, picograms per millilitre; IL-6, Interleukin 6.

**eFigure 4. Association of BMI deciles with incident type 2 diabetes in EPIC-Norfolk.** The association of BMI deciles with incident type 2 diabetes was estimated using multivariable Cox regression in the EPIC-Norfolk study. Age, sex, IL-6 levels, WHR, ethnicity, education level, family history of type 2 diabetes, smoking status, average physical activity per week, and average units of alcohol per week were covariates in the models. Hazard ratios for type 2 diabetes were estimated per kg/m^2^ higher BMI, relative to the lowest decile.

Abbreviations: HR, Hazard ratio; CI, Confidence interval; BMI, Body mass index; WHR, Waist-to-hip ratio; IL-6, Interleukin 6.

**eFigure 5. Association of Asp358Ala with type 2 diabetes and coronary artery disease by genotype in UK Biobank.** Type 2 diabetes results are shown in blue and those of coronary artery disease in orange. Multivariable logistic regression models were adjusted for age, sex, genotyping chip, the first 40 principal components and a kinship matrix. Odds ratios were estimated per copy of Asp358Ala relative to the wild-type Asp/Asp genotype. No statistical evidence of deviation from an additive association was observed for type 2 diabetes (P_non-linearity_ = 0·13) or coronary artery disease (P_non-linearity_ = 0·84).

Abbreviations: T2D, Type 2 diabetes; CAD, Coronary artery disease; OR, Odds ratio; CI, Confidence interval; Asp, Aspartic acid; Ala, Alanine.

**eFigure 6. Association of Asp358Ala with type 1 diabetes.**

In UK Biobank, the association of Asp358Ala with type 1 diabetes was estimated using multivariable logistic regression adjusted for age, sex, genotyping chip, the first 40 principal components and a kinship matrix. The result was meta-analysed with summary statistics from the type 1 diabetes genetics consortium and the Million Veteran Program respectively. Odds ratios for type 1 diabetes were estimated per copy of Asp358Ala. It should be noted that despite the use of the largest available studies and the sufficient power of this analysis, there may too few studies to make definitive statistical conclusions of the association between Asp358Ala and type 1 diabetes.

Abbreviations: OR, Odds ratio; CI, Confidence interval; Asp, Aspartic acid; Ala, Alanine.

**eFigure 7. Excluding type 1 diabetes cases using a 29-variant polygenic risk score. Panel A.** Theoretical distribution of the polygenic score in diabetes cases·^8^ Type 2 diabetes cases are shown in blue and type 1 diabetes cases in red. A z-score of 0·053 (actual value in UK Biobank, 13·04) denotes the median of the overall diabetes distribution (**Table 2**). **Panels B and C** show the number and percentage, respectively, of type 1 diabetes cases misclassified as type 2 diabetes that would be included in the analysis before and after applying the polygenic score exclusion. These simulations assume a 10% prevalence of type 1 diabetes amongst people with diabetes in the UK Biobank study^8^ and simulate percentage misclassification ranging from 100% to 0%.

Abbreviations: T1D, Type 1 diabetes; T2D, Type 2 diabetes.

**eFigure 8. Association of Asp358Ala with continuous metabolic traits.**

A Bonferroni corrected significance threshold of P<·005 (·05 / 10 continuous traits) was used.

Abbreviations: HbA1c, glycated haemoglobin; BMI, Body mass index; WHR, Waist-to-hip ratio; WHRadjBMI, WHR adjusted for BMI; mmol, millimoles; L, Litre; pmol, picomoles; Kg, Kilograms; m, meters; cm, centimetres Asp, Aspartic acid; Ala, Alanine; N, Number of participants.

**eFigure 9. Interaction of Asp358Ala with type 2 diabetes genetic risk factors on type 2 diabetes. Panel a.** The association of *IL6R* Asp358Ala with type 2 diabetes was estimated in individuals above or below the median value for 10 polygenic scores capturing genetic predisposition to risk factors for type 2 diabetes. The association with type 2 diabetes from the main analysis is shown below each score for comparison. A Bonferroni corrected significance threshold of P<·005 (·05/10 tests for heterogeneity) for the heterogeneity P-value was used. **Panel b.** The association of *IL6R* Asp358Ala with HbA1c levels was estimated in individuals above or below the median value for a polygenic score capturing genetic predisposition to higher BMI. The association with HbA1c in UK Biobank is shown for comparison. Estimates for HbA1c are expressed in % per copy of Asp358Ala.

Abbreviations: HbA1c, glycated haemoglobin; SNPs, Single nucleotide polymorphisms; BMI, Body mass index; adj., adjusted for; GoF, Gain-of-function; OR, Odds ratio

**eFigure 10. Projection of the range of possible effects of *IL6R* blocking therapy on the risk of type 2 diabetes in a primary prevention setting.** The ratio between lifetime exposure (genetic association) to short-term exposure (clinical trial) to lower IL-6R-mediated inflammation is on the x-axis. Lower ratio values correspond to estimates of longer treatment duration. The projected % reduction in diabetes risk, projected based on data from clinical trials of different dosages of tocilizumab, is represented on the y-axis.

Abbreviations: CI, Confidence interval; mg, milligrams; kg, kilograms

**eFigure 11. Funnel plot for publication bias assessment.** Illustration of the relationship between the hazard ratio for type 2 diabetes per log-pg/mL higher IL-6 levels and standard error for each study in included in the systematic review of prospective studies. Each study is represented by a dot and the estimated hazard ratio is depicted by the red line. The 95% confidence interval is shown by the grey contours.

Abbreviations: CI, Confidence interval

**References**

1 Wang X, Bao W, Liu J, Ouyang Y-Y, Wang D, Rong S *et al.* Inflammatory markers and risk of type 2 diabetes: a systematic review and meta-analysis. *Diabetes Care* 2013; **36**: 166–75.

2 Dallmeier D, Larson MG, Wang N, Fontes JD, Benjamin EJ, Fox CS. Addition of Inflammatory Biomarkers Did Not Improve Diabetes Prediction in the Community: The Framingham Heart Study. *J Am Heart Assoc* 2012; **1**: e000869–e000869.

3 Marques-Vidal P, Schmid R, Bochud M, Bastardot F, von Känel R, Paccaud F *et al.* Adipocytokines, Hepatic and Inflammatory Biomarkers and Incidence of Type 2 Diabetes. The CoLaus Study. *PLoS One* 2012; **7**: e51768.

4 Koloverou E, Panagiotakos DB, Georgousopoulou EN, Chrysohoou C, Tousoulis D, Stefanadis C *et al.* Single and combined effects of inflammatory markers on 10 year diabetes incidence: The mediating role of adiposity—Results from the ATTICA cohort study. *Diabetes Metab Res Rev* 2018; **34**: e2939.

5 Egger M, Smith GD, Phillips AN. Meta-analysis: Principles and procedures. Br. Med. J. 1997; **315**: 1533–1537.

6 Lotta LA, Wittemans LBL, Zuber V, Stewart ID, Sharp SJ, Luan J *et al.* Association of Genetic Variants Related to Gluteofemoral vs Abdominal Fat Distribution With Type 2 Diabetes, Coronary Disease, and Cardiovascular Risk Factors. *JAMA* 2018; **320**: 2553.

7 Bulik-Sullivan BK, Loh P-R, Finucane HK, Ripke S, Yang J, Patterson N *et al.* LD Score regression distinguishes confounding from polygenicity in genome-wide association studies. *Nat Genet* 2015; **47**: 291–295.

8 Thomas NJ, Jones SE, Weedon MN, Shields BM, Oram RA, Hattersley AT. Frequency and phenotype of type 1 diabetes in the first six decades of life: A cross-sectional, genetically stratified survival analysis from UK Biobank. *Lancet Diabetes Endocrinol* 2017; **6**: 122–129.

9 Ferreira RC, Freitag DF, Cutler AJ, Howson JMM, Rainbow DB, Smyth DJ *et al.* Functional IL6R 358Ala Allele Impairs Classical IL-6 Receptor Signaling and Influences Risk of Diverse Inflammatory Diseases. *PLoS Genet* 2013; **9**: e1003444.

10 Sarwar N, Butterworth AS, Freitag DF, Gregson J, Willeit P, Gorman DN *et al.* Interleukin-6 receptor pathways in coronary heart disease: A collaborative meta-analysis of 82 studies. *Lancet* 2012; **379**: 1205–1213.

11 Swerdlow DI, Holmes M V., Kuchenbaecker KB, Engmann JEL, Shah T, Sofat R *et al.* The interleukin-6 receptor as a target for prevention of coronary heart disease: A mendelian randomisation analysis. *Lancet* 2012; **379**: 1214–1224.

12 Burgess S, Ference BA, Staley JR, Freitag DF, Mason AM, Nielsen SF *et al.* Association of LPA Variants With Risk of Coronary Disease and the Implications for Lipoprotein(a)-Lowering Therapies: A Mendelian Randomization Analysis. *JAMA Cardiol* 2018. doi:10.1001/JAMACARDIO.2018.1470.

13 Ference BA, Kastelein JJP, Ray KK, Ginsberg HN, Chapman MJ, Packard CJ *et al.* Association of Triglyceride-Lowering LPL Variants and LDL-C-Lowering LDLR Variants with Risk of Coronary Heart Disease. *JAMA - J Am Med Assoc* 2019; **321**: 364–373.

14 Locke AE, Kahali B, Berndt SI, Justice AE, Pers TH, Day FR *et al.* Genetic studies of body mass index yield new insights for obesity biology. *Nature* 2015; **518**: 197–206.

15 Wheeler E, Leong A, Liu CT, Hivert MF, Strawbridge RJ, Podmore C *et al.* Impact of common genetic determinants of Hemoglobin A1c on type 2 diabetes risk and diagnosis in ancestrally diverse populations: A transethnic genome-wide meta-analysis. *PLoS Med* 2017; **14**: e1002383.

16 Manning AK, Hivert M-F, Scott RA, Grimsby JL, Bouatia-Naji N, Chen H *et al.* A genome-wide approach accounting for body mass index identifies genetic variants influencing fasting glycemic traits and insulin resistance. *Nat Genet* 2012; **44**: 659–669.

17 Scott RA, Lagou V, Welch RP, Wheeler E, Montasser ME, Luan J *et al.* Large-scale association analyses identify new loci influencing glycemic traits and provide insight into the underlying biological pathways. *Nat Genet* 2012; **44**: 991–1005.

18 Scott RA, Fall T, Pasko D, Barker A, Sharp SJ, Arriola L *et al.* Common genetic variants highlight the role of insulin resistance and body fat distribution in type 2 diabetes, independent of obesity. *Diabetes* 2014; **63**: 4378–4387.

19 Lotta LA, Mokrosiński J, Mendes de Oliveira E, Li C, Sharp SJ, Luan J *et al.* Human Gain-of-Function MC4R Variants Show Signaling Bias and Protect against Obesity. *Cell* 2019; **177**: 597-607.e9.
